# Dopamine D1 Receptor Contributes to Glucocorticoid‐Associated Osteonecrosis of Femoral Head Protection Through the ATF3/CHOP Axis to Inhibit Osteoblastic Apoptosis

**DOI:** 10.1002/advs.202502276

**Published:** 2025-06-29

**Authors:** Kai Zheng, Wenming Li, Tianhao Wang, Gaoran Ge, Wei Zhang, Yi Qin, Wenhao Li, Zebin Wu, Zhen Wang, Gang Rui, Yaozeng Xu, Dechun Geng

**Affiliations:** ^1^ Department of Orthopedics The First Affiliated Hospital of Soochow University Suzhou 215000 China; ^2^ Department of Orthopedics The First Affiliated Hospital of Xiamen University School of Medicine Xiamen University Xiamen 361000 China; ^3^ Department of Orthopedics Wuxi Ninth People's Hospital Affiliated to Soochow University Wuxi 214000 China; ^4^ Department of Orthopedics Suzhou Kowloon Hospital Shanghai Jiao Tong University School of Medicine Suzhou Jiangsu 215000 China

**Keywords:** apoptosis, dopamine D1 receptors, glucocorticoid‐associated ONFH, osteogenesis

## Abstract

The nervous system plays a pivotal regulatory role in the maintenance of bone homeostasis, and the protective effects of dopamine and its receptors on bone metabolism are emerging. Despite these protective roles, the functional contribution of dopaminergic signaling, particularly through specific receptor subtypes, remains unexplored in glucocorticoid (GC)‐associated osteonecrosis of the femoral head (ONFH) pathogenesis. Here, the dopamine D1 receptor (DRD1), a G protein‐coupled receptor with few identified bone‐related functions, is identified as a positive regulator of GC‐induced apoptosis. The dopamine levels in the serum of GC‐associated ONFH patients are significantly lower than those in the normal population. The protein and gene expression levels of DRD1 and the number of DRD1‐positive cells are abnormally elevated in the pathological state of GC‐associated ONFH, and DRD1 is expressed in osteoblasts. Overexpression of DRD1 attenuates GC‐induced osteogenic inhibition and apoptosis in vivo and in vitro. Mechanistically, overexpression of DRD1 elevates cAMP levels, activates downstream protein kinase A, and inhibits GC‐induced endoplasmic reticulum stress and apoptosis through the ATF3/CHOP signaling pathway, thus improving bone homeostasis. Importantly, Madopar, an FDA‐approved dopaminergic agent, inhibits GC‐induced osteoblastic apoptosis and ONFH via DRD1. Collectively, this study not only deciphers a previously unrecognized DRD1‐mediated neuro‐osteogenic axis but also repurposes an FDA‐approved drug (Madopar) for precision ONFH management.

## Graphical abstract

This work identifies a novel mechanism by which dopamine D1 receptor (DRD1) contributes to the pathogenesis of glucocorticoid (GC)‐associated osteonecrosis of the femoral head (ONFH) through the regulation of osteoblastic apoptosis, indicating that DRD1 serves as a critical mediator of the crosstalk between the nervous and skeletal systems. This work provides groundbreaking insights into DRD1's role in GC‐associated ONFH and offers a compelling case for repurposing Madopar.

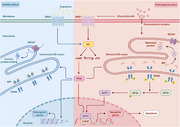

## 1 Introduction

Osteonecrosis of the femoral head (ONFH) is a severe clinical consequence of glucocorticoid (GC) therapy characterized by bone microarchitecture deterioration, circulatory disruption in weight‐bearing areas, and subsequent hip degeneration.^[^ ^1^ ^]^ This condition disproportionately affects younger adults, with an estimated 20 million annual cases worldwide, imposing a substantial burden on healthcare systems.^[^ ^2^, ^3^ ^]^ Current therapeutic paradigms remain limited to palliative surgical interventions.^[^ ^4^ ^]^ However, the efficacy of hip preservation surgery varies from 40% to 80%, while total hip arthroplasty achieves 90% 10‐year survivorship, and still presents suboptimal outcomes for active populations.^[^ ^5^, ^6^ ^]^ The critical unmet need lies in developing targeted early pharmacotherapies addressing GC‐associated ONFH pathogenesis.

The pathophysiology of GC‐associated ONFH involves complex dysregulation of bone remodeling homeostasis. While osteoclast suppression mitigates bone resorption, femoral head structural integrity fundamentally depends on osteoblastic viability and micro‐fracture repair capacity. Excessive use of GC‐induced osteoblast apoptosis is known to be one of the primary pathological changes caused by direct hormonal effects on bone cells.^[^ ^7^ ^]^ GC excess induces osteoblastic apoptosis via dual principal pathways: 1) rapid activation of the proline‐rich tyrosine kinase 2 (Pyk2)/c‐Jun N‐terminal kinase (JNK) cascade, leading to reactive oxygen species (ROS) accumulation;^[^ ^8^, ^9^ ^]^ 2) Activating transcription factor 4 (ATF4)/C/EBP‐homologous protein (CHOP)‐dependent endoplasmic reticulum stress (ERS) triggered by the unfolded protein response (UPR).^[^ ^10^, ^11^ ^]^ These events disrupt bone remodeling equilibrium, ultimately causing femoral head collapse. The role of GC‐induced osteoblast apoptosis in ONFH has been extensively investigated, however, its regulatory mechanisms still remain elusive.

Emerging evidence in neuroendocrinology reveals promising therapeutic strategies for targeting osteoblast apoptosis.^[^ ^12^ ^]^ The bidirectional communication between the skeletal and nervous systems provides a fine control of bone homeostasis.^[^ ^13^ ^]^ Our prior study identified cannabinoid receptor 2 is extensively expressed in bone cells and tissues, where it acts as a positive regulator of osteogenic activation and circulatory maintenance via the glycogen synthase kinase‐3β (GSK‐3β)/β‐catenin pathway.^[^ ^14^ ^]^ Furthermore, we demonstrated GC‐mediated upregulation of monoacylglycerol lipase (MAGL), a key enzyme of the endocannabinoid system in osteoblasts, whose inhibition attenuates GC‐induced apoptosis and oxidative stress via the kelch like ECH associated protein 1 (Keap1)/nuclear factor erythroid 2‐related factor 2 (Nrf2) signaling.^[^ ^15^ ^]^ Interestingly, cannabinoid‐dopamine interactions have been shown to be involved in physiology and pathophysiology through direct mechanisms or indirect mechanisms involving gamma‐aminobutyric acid (GABA) and glutamate neurons.^[^ ^16^, ^17^ ^]^ Therefore, identifying new neuroendocrine mechanisms involved in bone metabolism may uncover novel therapeutic strategies for GC‐associated ONFH.

Dopaminergic signaling exerts pleiotropic effects on skeletal physiology beyond its canonical neurological functions through five G protein‐coupled dopamine (DA) receptor subtypes^[^ ^18^, ^19^ ^]^ DA receptors are functionally categorized into D1‐like (D1, D5) and D2‐like (D2, D3, D4) families with distinct expression patterns.^[^ ^20^, ^21^ ^]^ Notably, DA receptors are abundantly expressed in osteoclasts and osteoblasts.^[^ ^22^ ^]^ Moreover, dopamine transporter gene‐deficient (DAT‐/‐) mice exhibit profound bone histomorphometry and biomechanical abnormalities, underscoring a strong connection between DA receptors and bone homeostasis.^[^ ^23^ ^]^ Schwendich et al. found that dopamine activates dopamine D2 receptors (DRD2) and significantly increases bone mineralization in osteoblasts without altering the expression of osteoclast markers in arthritis.^[^ ^24^ ^]^ In addition, DA promotes osteoblast differentiation in a DRD1‐dependent manner via the ERK1/2 signaling pathway.^[^ ^25^ ^]^ While multiple DA receptor subtypes exist, DRD1 has shown unique regulatory potential in bone metabolism, warranting focused investigation.

**Scheme 1:**
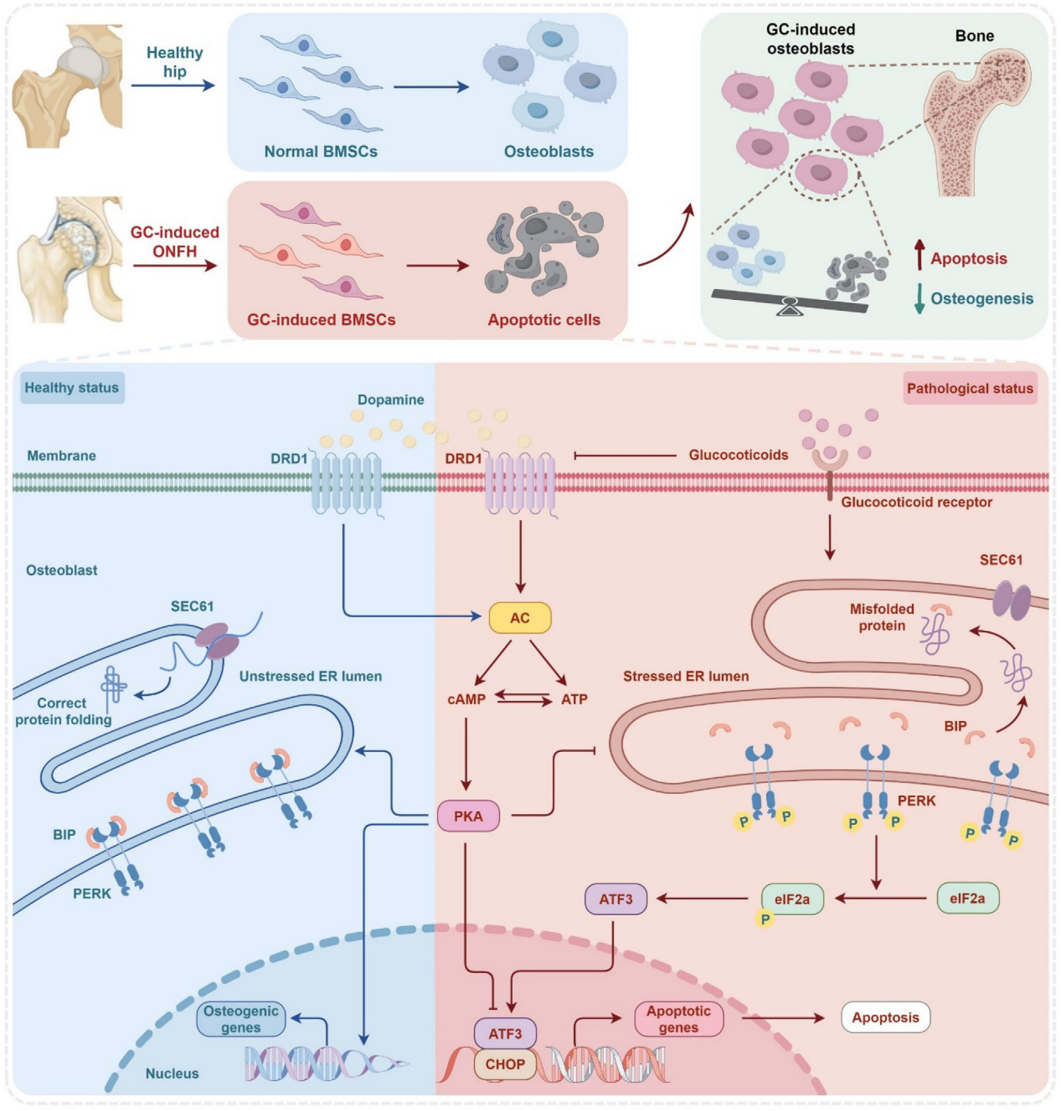
Schematic illustration of the effect and mechanism of DRD1 in GC‐associated ONFH.

Herein, we propose that the dopamine levels in the serum of GC‐associated ONFH patients are significantly lower than those in the normal population. The protein and gene levels of DRD1 and the number of DRD1‐positive cells are aberrantly elevated in pathological tissues. Mechanistically, DRD1 upregulation of activates cyclic adenosine monophosphate (cAMP) levels and downstream protein kinase A to attenuate GC‐induced endoplasmic reticulum stress and apoptosis through inhibition of the ATF3/CHOP signaling pathway, thus improving bone homeostasis (**Scheme** **1**). We also validate the therapeutic efficacy of Madopar (levodopa/benserazide), an established dopaminergic formulation, in preventing GC‐associated ONFH progression through DRD1‐dependent mechanisms. These findings establish DRD1 as both a novel diagnostic biomarker and therapeutic target while providing clinical translation rationale for neuromodulatory agents in ONFH management.

## 2 Results

### 2.1 GC Abnormally Alters DRD1 Expression In Vivo and In Vitro

**Figure 1:**
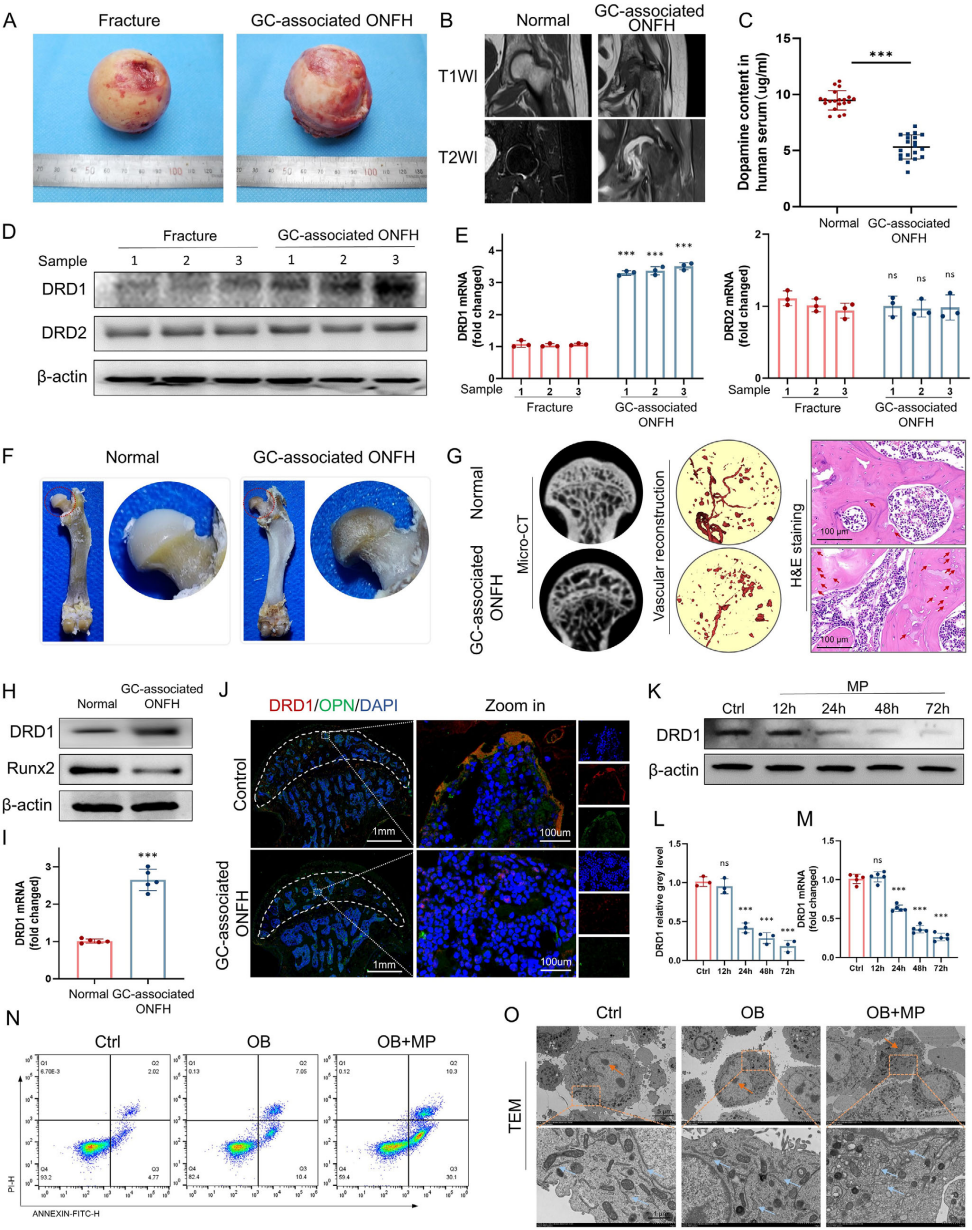
GC abnormally alters DRD1 expression in vivo and in vitro. A) Gross appearance of femoral heads from patients diagnosed with GC‐associated ONFH and femoral neck fracture. B) The representative T1 and T2‐weighted MRI images. C) ELISA of DA concentration in human serum (*n* = 20 per group). D) Western blot of DRD1, DRD2, and β‐actin in different clinical samples (*n* = 3 per group). E) Gene levels of DRD1 and DRD2 (*n* = 3 per group). F) Gross appearance of femoral heads from GC‐associated ONFH rat and normal rat. G) Representative images of Micro‐CT, vascular reconstruction, and H&E staining in different animal samples. H) Western blot of DRD1, Runx2, and β‐actin in different animal samples (*n* = 5 per group). I) Gene levels of DRD1 (*n* = 5 per group). J) Immunofluorescence staining of rat femoral head sections with anti‐DRD1 and OPN antibodies. K) Western blot of DRD1 and β‐actin in osteoblasts treated with MP at different time points (*n* = 3 per group). L) Semiquantitative analysis of the DRD1 protein levels in different samples. M) Gene levels of DRD1 (*n* = 5 per group). N) Flow cytometric analysis of apoptosis in BMSCs, osteogenesis‐induced BMSCs, and MP‐stimulated osteogenesis‐induced BMSCs. O) Representative images of TEM of those cells (*n* = 3 per group, ns denotes not significant, ^***^ denotes *p* < 0.001 compared to the normal, fracture, or control group).

To determine the role of DA and its receptors in GC‐associated ONFH, clinical samples were collected from patients diagnosed with femoral neck fracture and GC‐associated ONFH (**Table** **1**; Table S1, Supporting Information). The GC‐associated ONFH samples showed severe abnormalities in gross appearance (**Figure** **1**A). The T1 and T2‐weighted MRI images showed focal necrosis in the weight‐bearing zone of the femoral head and the dimensions of necrosis was 4.0 × 7.0 × 2.1cm^3^ (Figure 1B). The DA levels in the serum of GC‐associated ONFH patients were significantly lower than those in the healthy controls (Figure 1C), suggesting a potential involvement of DA receptors in dopaminergic signaling pathways in the pathogenesis of GC‐associated ONFH. To identify which DA receptor was involved in GC‐associated ONFH, proteins, and RNA were extracted from femoral head tissues. The protein and gene expression levels of DRD1 were significantly higher in GC‐associated ONFH samples than in femoral neck fracture samples (Figure 1D,E; Figure S1, Supporting Information). However, changes in the expression of DRD2 at the tissue protein and gene levels were not obvious.

**Table 1:** Basic patient characteristics.

Variables	GC‐associated ONFH						
	No.1	No.2	No.3	No.4	No.5	No.6	Summary
Sex	female	male	female	female	female	female	1 M/5 F
Age (yrs)	47	55	53	49	56	59	53.17 ± 4.49
BMI (kg m^−2^)	26.56	32.39	24.44	25.89	22.77	29.78	26.97 ± 3.54
Side	left	left	right	left	right	left	4 L/2 R
Ficat stage	IV	IV	IV	IV	IV	IV	6 IV

	Femoral neck fracture						
	No.1	No.2	No.3	No.4	No.5	No.6	Summary
Sex	male	female	female	male	female	female	2 M/4 F
Age (yrs)	48	51	56	57	54	60	54.33 ± 4.32
BMI (kg m^−2^)	24.22	27.77	19.33	29.69	25.39	27.59	25.66 ± 3.65
Side	right	left	right	left	left	right	3 R/3 L
Garden classification	IV	III	IV	IV	IV	III	2 III/ 4 IV

A rat model of GC‐associated ONFH was established, characterized by an abnormally red coloration and a rough surface in the weight‐bearing area of the femoral head. In contrast, the femoral head of the control group was white in color with a smooth surface and intact cartilage (Figure 1F). Moreover, noticeably sparse bone trabeculae, reduced vascularization, and abundant necrotic bone cells can be seen in the femoral head of GC‐associated ONFH rats (Figure 1G). An increase in the number of DRD1‐positive cells was observed in GC‐associated ONFH rats compared to controls, especially in cartilage, but lower expression of DRD1 was observed in bone marrow in GC‐associated ONFH rats (Figure S2, Supporting Information). Consistently, the protein and gene levels of DRD1 were increased, while osteogenesis‐related runt‐related transcription factor 2 (Runx2) expression was suppressed in GC‐associated ONFH rats (Figure 1H,I; Figure S3, Supporting Information). To identify that DRD1 shows selective expression in osteoblasts, colocalization of DRD1 and osteoblast marker osteopontin (OPN) was evaluated in the GC‐associated ONFH and control group. The immunofluorescence staining showed that the fluorescence sites of DRD1 (red) and osteopontin (OPN, green) mostly overlap, confirming DRD1 expression in osteoblasts residing on the trabecular bone surface in the epiphysis of the femoral head (Figure 1J).

How GC affects the expression of DRD1 in osteoblasts was further evaluated. The bone marrow mesenchymal stem cells (BMSCs) were isolated and identified by flow cytometry (Figure S4, Supporting Information). BMSCs viability following exposure to varying concentrations of methylprednisolone (MP) for 1, 4, and 7 days was assessed using a cell counting kit‐8 (CCK‐8) assay. No significant cytotoxicity was detected at MP concentrations below 10 µm (Figure S5, Supporting Information). Exposure to MP over 24 h resulted in a significant time‐dependent decrease in the protein and gene expression levels of DRD1 in osteoblasts (Figure 1K–M). Notably, the discrepancy between in vitro and in vivo DRD1 expression patterns may be attributed to the distinct cell type‐specific expression, localization, cell‐nonautonomous mechanism, and differences in microenvironmental crosstalk.^[^ ^26^, ^27^ ^]^ These results indicate that DRD1 may play a role in GC‐associated ONFH.

The differentiation and function of osteoblasts under MP condition was also examined. A decrease in alkaline phosphatase (ALP) activity and mineralization capacity was observed in BMSCs incubated with MP (Figure S6, Supporting Information). Consistently, the expression of characteristic osteoblast proteins and transcripts, including Osterix and osteocalcin (OCN) was significantly reduced following MP treatment (Figure S7, Supporting Information). Conversely, expression levels of apoptotic markers, including BCL‐2‐associated X protein (BAX) and cleaved‐caspase 3 were up‐regulated. In addition, flow cytometry analysis revealed that the number of apoptotic osteoblasts induced by MP significantly increased, especially the percentage of early apoptotic cells, which increased from 10.4% by osteogenic induction to 30.1% by osteogenic induction and MP (Figure 1N). The transmission electron microscopy (TEM) observation revealed an early manifestation of apoptotic osteoblasts, presented with condensed chromatin and pyknotic nuclei (Figure 1O). In addition, the endoplasmic reticulum (ER) became swollen, rough, and fragmented. These results suggest that GC induces osteogenic inhibition and enhances ER stress and early apoptosis in vitro.

### 2.2 Overexpression of DRD1 Inhibits GC‐Induced Osteoblastic Apoptosis

**Figure 2:**
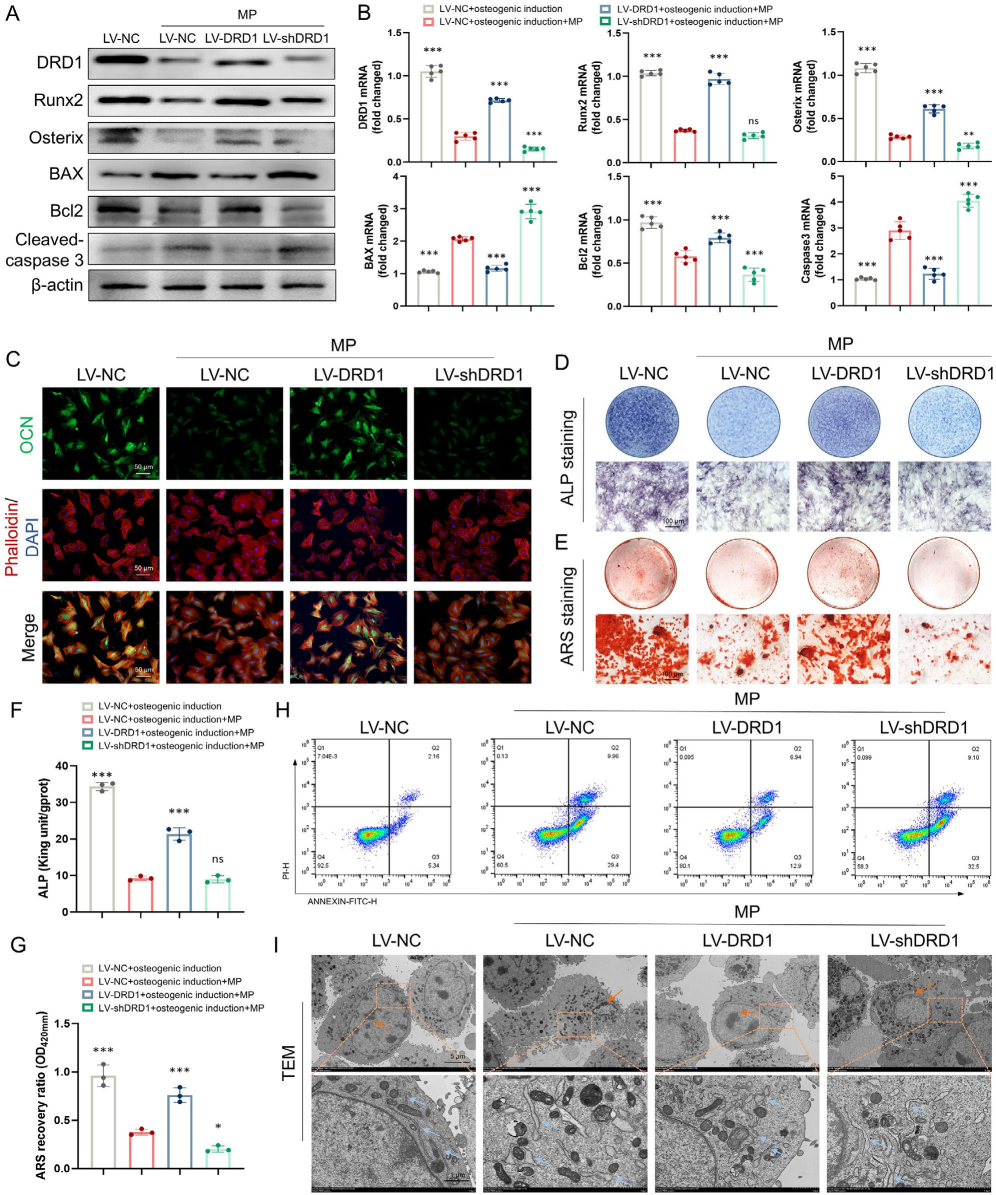
Overexpression of DRD1 inhibits GC‐induced osteoblastic apoptosis. A) Western blotting of DRD1, Runx2, Osterix, BAX, Bcl2, cleaved‐caspase3, and β‐actin in osteogenesis‐induced BMSCs treated with LV‐NC, LV‐DRD1, LV‐shDRD1 and then stimulated with MP (*n* = 3 per group). B) Gene levels of DRD1, Runx2, Osterix, BAX, Bcl2, and Caspase3 in those cells (*n* = 5 per group). C) Immunofluorescence staining of OCN (green) in those cells. D) ALP staining of those cells. E) Alizarin red S staining of those cells. F) Quantitative evaluation of ALP activity (*n* = 3 per group). G) Semiquantitative evaluation of ECM mineralization (*n* = 3 per group). H) Flow cytometric analysis of apoptosis in those cells. I) Representative images of TEM of those cells (ns denotes not significant, ^*^ denotes *p* < 0.05, ^**^ denotes *p* < 0.01, ^***^ denotes *p* < 0.001 compared to the LV‐NC + osteogenic induction + MP group).

To gain more insight into the effect of DRD1 on GC‐induced osteoblastic apoptosis, DRD1 was overexpressed and silenced by lentivirus in BMSCs. The infection efficiency exceeded 80% (Figure S8A,B, Supporting Information). The feasibility of regulating the expression of DRD1 via LV‐DRD1 and LV‐shDRD1 was validated at the protein and gene levels (Figure S8C–E, Supporting Information). Overexpression of DRD1 in osteogenesis‐induced BMSCs effectively rescued GC‐induced suppression of osteogenesis and reduced apoptosis, whereas DRD1 knockdown exerted the opposite effect (**Figure** **2**A; Figure S9, Supporting Information). These osteogenic and apoptotic markers showed corresponding trends at the transcript level (Figure 2B). Furthermore, the fluorescence intensity of OCN in the plasma membrane was increased by LV‐DRD1 under MP intervention (Figure 2C). DRD1 overexpression enhanced ALP activity and mineralization capacity in MP‐treated cells, while inhibition further suppressed the deposition of mineralized extracellular matrix (Figure 2D–G).

The inhibitory effect of DRD1 on GC‐induced osteoblastic apoptosis was also examined. The number of apoptotic osteoblasts induced by MP was significantly decreased by LV‐DRD1, especially the percentage of early apoptotic cells, which decreased from 29.4% by LV‐NC treatment to 12.9% by LV‐DRD1 treatment (Figure 2H). Similarly, decreased chromatin condensation and margination with less swollen and fragmented ERs were indicated by TEM observation of osteoblasts treated with MP and LV‐DRD1 (Figure 2I). These results suggest that DRD1 overexpression inhibits GC‐induced ER stress and early osteoblastic apoptosis. These positive regulatory effects of DRD1 are exerted without additional DA in vitro, thus the dopamine concentration in the culture medium was detected (Figure S10, Supporting Information). Extremely low concentrations of DA can be detected in osteogenic culture medium supernatants from osteoblasts. This result proposes that the protect effect of DRD1 may be activated in low dopamine environments.

### 2.3 Overexpression of DRD1 Ameliorates GC‐Associated ONFH

**Figure 3:**
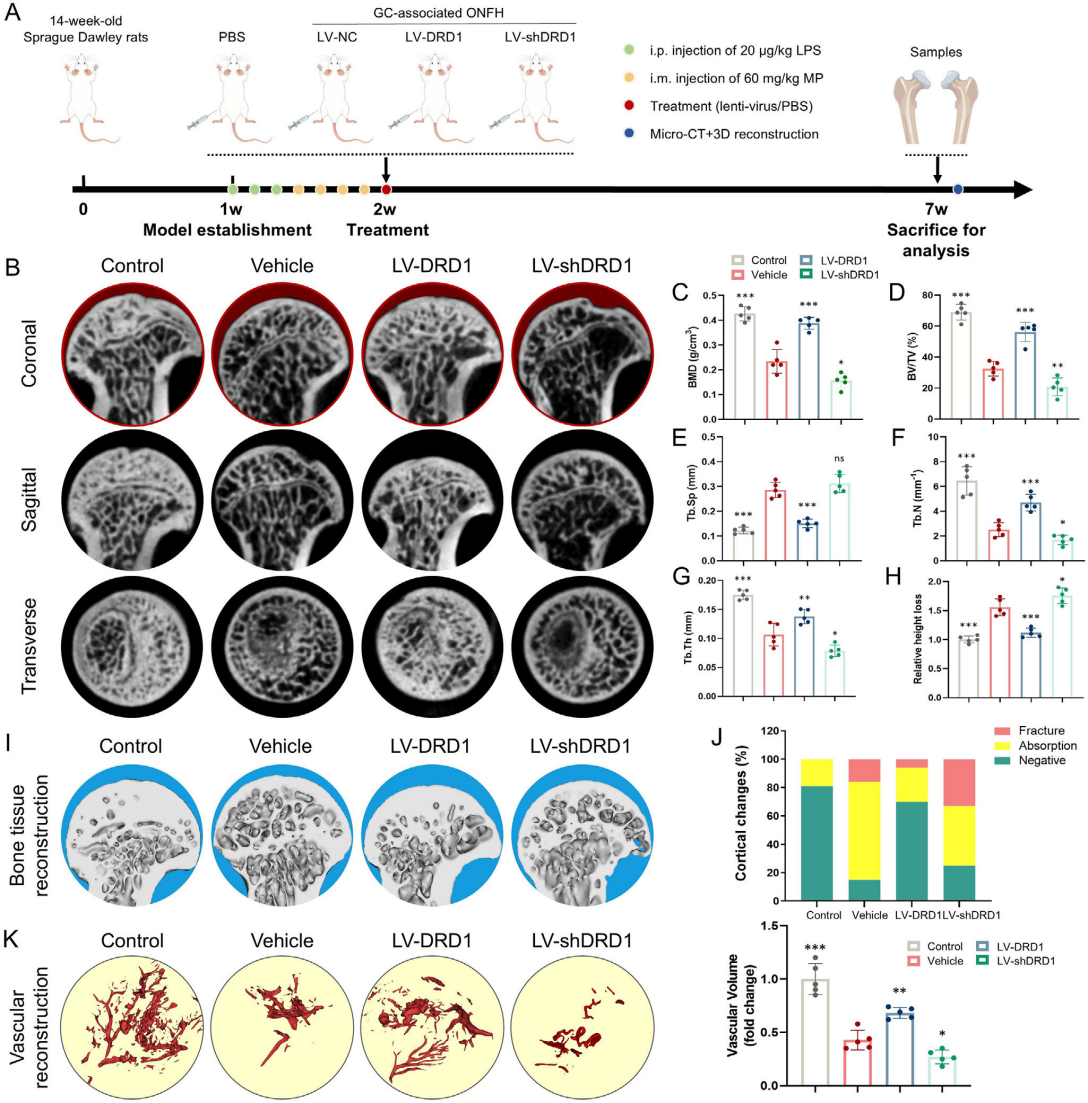
In vivo evaluation of GC‐associated ONFH alleviation after different treatments. A) Schematic illustration of the animal experiments. B) Representative micro‐CT images of GC‐associated ONFH in coronal, sagittal, and transverse planes (red shadow indicates femoral head relative height loss). C) BMD (g/mm^3^). D) BV/TV (%). E) Tb.Sp (mm). F) Tb.N (mm^−1^). G) Tb.Th (mm). H) Relative height loss. I) Representative images of 3D reconstruction of the femoral head. J) Cortical changes. K) Vascular reconstruction of the femoral head and quantitative analysis of vascular volume (*n* = 5 per group, ns denotes not significant, ^*^ denotes *p* < 0.05, ^**^ denotes *p* < 0.01, ^***^ denotes *p* < 0.001 compared to the vehicle group).

To assess the therapeutic potential of DRD1 in GC‐associated ONFH, we tested its effects on pathological changes in GC‐associated ONFH and related bone parameters in a rat model (**Figure** **3**A). Total protein and RNA were extracted from femoral heads following lentivirus treatment. The expression of DRD1 was significantly upregulated by LV‐DRD1 and downregulated by LV‐shDRD1 at both protein and gene levels (Figure S11, Supporting Information). Micro‐CT imaging revealed that DRD1 overexpression mitigated radiographic changes in GC‐associated ONFH. The vehicle group exhibited abnormal femoral head morphology, significant trabecular bone loss, and collapse in the weight‐bearing region, as seen in coronal, sagittal, and transverse views (Figure 3B). LV‐DRD1 treatment effectively attenuated these pathological manifestations, whereas LV‐shDRD1 treatment exacerbated osteonecrosis. Quantitative analysis revealed that the LV‐DRD1 group significantly increased bone volume per total volume (BV/TV), trabecular number (Tb. N), trabecular thickness (Tb. Th), mineral bone density (BMD) parameters while reducing trabecular separation (Tb. Sp) and relative height loss compared to the vehicle group (Figure 3C–H). And 3D reconstruction of Micro‐CT further confirmed that LV‐DRD1 treatment mitigated the pathological changes in cortical bone (Figure 3I,J). Additionally, vascular reconstruction in the femoral head was significantly enhanced by LV‐DRD1 treatment (Figure 3K). These findings suggest that DRD1 overexpression effectively attenuates the pathological manifestations in the cortical and weight‐bearing region of the femoral head.

**Figure 4:**
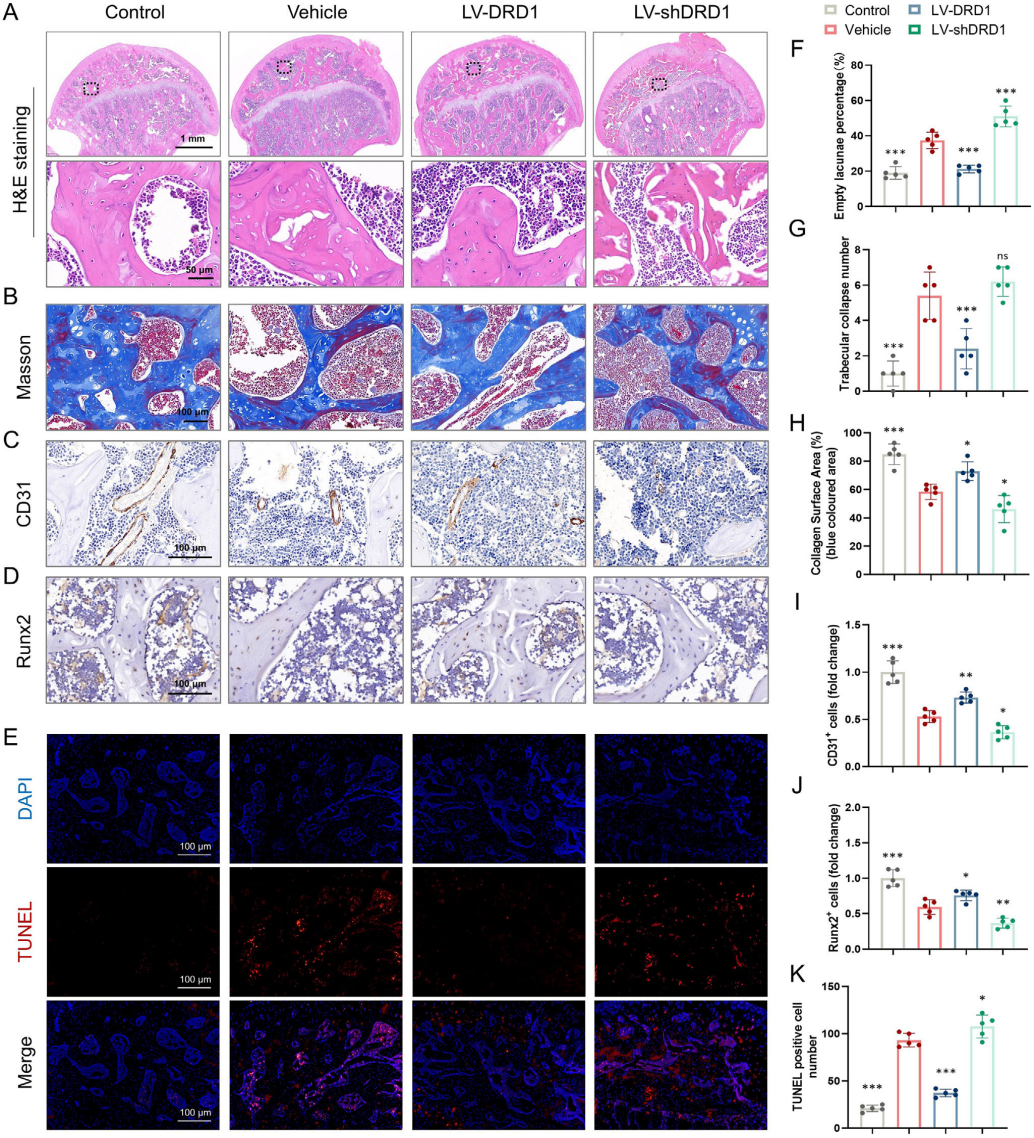
Histological staining and histomorphometric analysis of GC‐associated ONFH in rat model. A) Representative images of H&E staining. B) Representative images of Massson staining. C) IHC staining of CD31. D) IHC staining of DRD1. E) TUNEL staining. F) Empty lacunae percentage (%). G) Trabecular collapse number. H) Collagen surface area (%, blue‐colored area). I) CD31^+^ cells number. J) Runx2^+^ cells number. K) TUNEL positive cell number (*n* = 5 per group, ns denotes not significant, ^*^ denotes *p* < 0.05, ^**^ denotes *p* < 0.01, ^***^ denotes *p* < 0.001 compared to the vehicle group).

Histological changes in GC‐associated ONFH after different treatments were also assessed. H&E staining verified the attenuation of GC‐associated ONFH upon treatment with LV‐DRD1 (**Figure** **4**A). Decreases in empty lacunae percentage and trabecular collapse number were observed in the LV‐DRD1 group compared to the vehicle group. In addition, a significantly greater percentage of empty lacunae and lower BV/TV were detected in the LV‐shDRD1 group (Figure 4F,G). The calcein‐Alizarin red S double labeling showed that LV‐DRD1 treatment enhanced dynamic bone formation (Figure S12A, Supporting Information). An increase in mineralizing surface and mineral apposition rate was observed in the LV‐DRD1 group (Figure S12B, Supporting Information). Massson staining showed that LV‐DRD1 treatment enhanced the calcification of new bone tissue (blue‐stained) in the weight‐wearing region of the femoral head (Figure 4B,H). An increase in CD31‐ and Runx2‐positive cells was observed in the LV‐DRD1 group (Figure 4C,D,I,J). Furthermore, TUNEL staining showed a significant reduction in apoptotic cells in the LV‐DRD1 group, but an increase in the LV‐shDRD1 group (Figure 4E,K). Collectively, these results reveal that DRD1 overexpression alleviates pathological and histological changes in GC‐associated ONFH and related bone parameters.

### 2.4 DRD1 Inhibits GC‐Induced Osteoblastic Apoptosis via ATF3/CHOP Signaling

**Figure 5:**
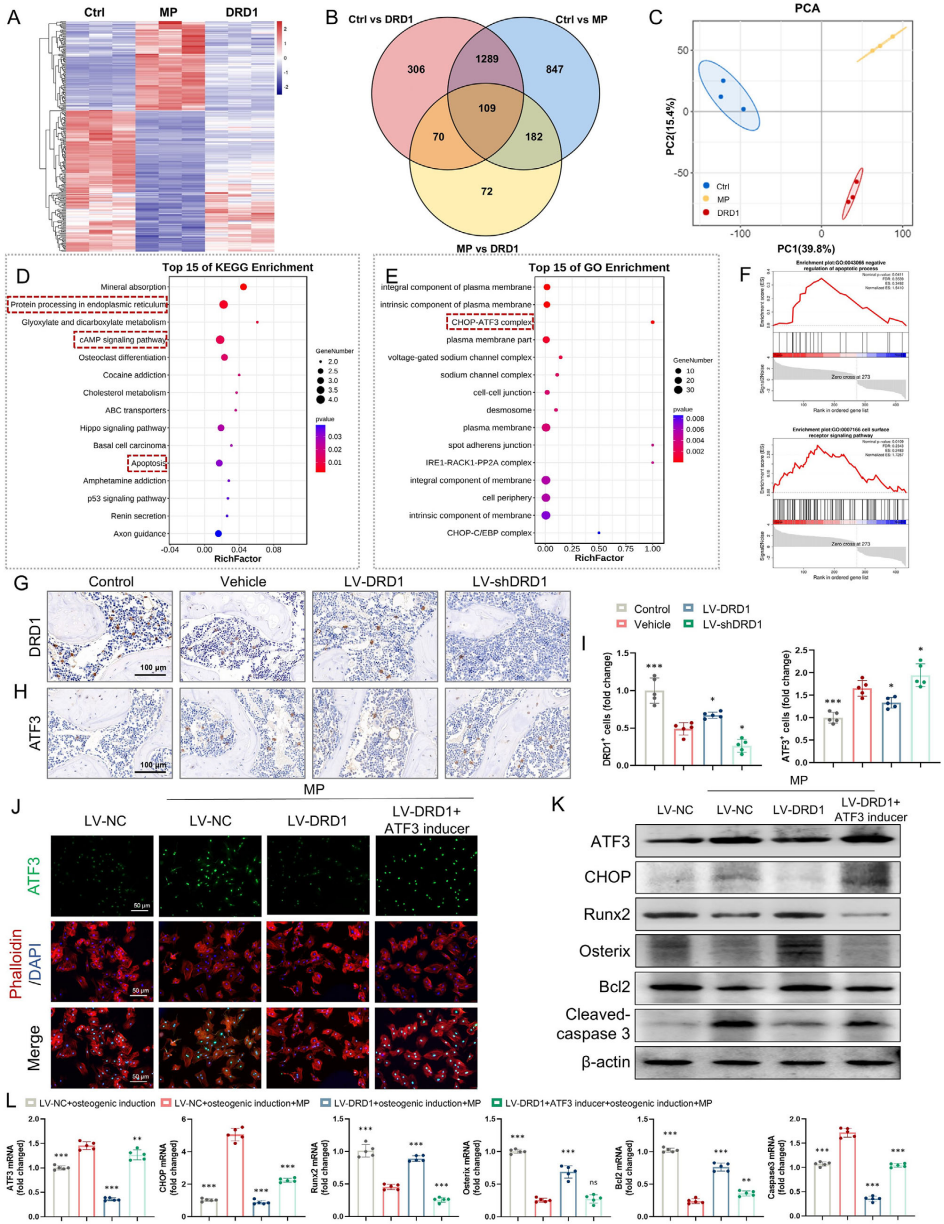
DRD1 inhibits GC‐induced osteoblastic apoptosis via ATF3/CHOP signaling. A) Heat map of DEGs in the control, MP, and MP+LV‐DRD1 groups. B) Venn diagram of the overlap of DEGs. C) PCA of the samples in three groups. D) KEGG enrichment analysis of DEGs. E) GO enrichment analysis of DEGs. F) GSEA analysis of related biological functions and pathways. G) IHC staining of DRD1 in the control, vehicle, LV‐DRD1, and LV‐shDRD1 groups. H) IHC staining of ATF3 in these four groups. I) DRD1^+^ and ATF3^+^ cells number (*n* = 5 per group). J) Immunofluorescence staining of ATF3 (green) in osteogenesis‐induced BMSCs treated with LV‐NC, LV‐DRD1, LV‐DRD1+ATF3 inducer and then stimulated with MP. K) Western blotting of ATF3, CHOP, Runx2, Osterix, Bcl2, cleaved‐caspase 3, and β‐actin in those cells. L) Gene levels of ATF3, CHOP, Runx2, Osterix, Bcl2 and Caspase 3 in those cells (*n* = 5 per group, ns denotes not significant, ^*^ denotes *p* < 0.05, ^**^ denotes *p* < 0.01, ^***^ denotes *p* < 0.001 compared to the LV‐NC + osteogenic induction + MP group).

To elucidate the mechanism by which DRD1 inhibits GC‐induced osteoblastic apoptosis, transcriptome sequencing was conducted using total RNA extracted from control, MP‐treated, and MP+LV‐DRD1‐treated groups. Volcano plots revealed 2427 differentially expressed genes (DEGs) between the control and MP groups, 1774 DEGs between the control and DRD1 groups, and 433 DEGs between the MP and DRD1 groups (Figure S13, Supporting Information). A heatmap visualized the gene expression patterns among the three groups (**Figure** **5**A), and a Venn diagram illustrated the overlap among DEGs (Figure 5B). Principal component analysis revealed a distinct distribution among the groups (Figure 5C). Kyoto encyclopedia of genes and genomes (KEGG) enrichment revealed that the MP+LV‐DRD1 group modulated pathways including protein processing in the endoplasmic reticulum, cAMP signaling, and apoptosis (Figure 5D). Gene ontology (GO) enrichment highlighted significant enrichment of the CHOP‐ATF3 complex, suggesting its role in regulating osteoblastic apoptosis (Figure 5E). Consistently, gene set enrichment analysis (GSEA) revealed that the MP+LV‐DRD1 group was positively associated with the negative regulation of the apoptotic process and cell surface receptor signaling pathway (Figure 5F). In addition, the local heat map showed upregulated expression of ATF3, Ddit3 (CHOP), growth arrest and DNA damage inducible alpha (Gadd45a), and endoplasmic reticulum to nucleus signalling 1 (Ern1) in the MP group, which was reversed by DRD1 overexpression (Figure S14A, Supporting Information). STRING analysis also confirmed protein‐protein interaction networks involving DRD1 and these genes (Figure S14B, Supporting Information). In addition, the in vivo changes of DRD1 and downstream ATF3 was validated by immunohistochemistry (IHC) staining in model animals (Figure 5G–I). The number of ATF3‐positive cells was decreased by LV‐DRD1, but increased by LV‐shDRD1. These results demonstrate that the ATF3/CHOP signaling pathway may be involved in the DRD1 regulation of GC‐induced osteoblastic apoptosis.

To further test whether the effect of DRD1 on GC‐induced osteoblastic apoptosis is dependent on the ATF3/CHOP signaling pathway, an ATF3 inducer was co‐administered with LV‐DRD1. Under MP treatment, LV‐DRD1 suppressed nuclear ATF3 fluorescence intensity, but this effect was reversed by co‐treatment with the ATF3 inducer (50 µm, Figure 5J). DRD1 overexpression also suppressed protein levels of CHOP, cleaved‐caspase 3, while enhancing Runx2, Osterix, and B‐cell lymphoma‐2 (Bcl2) (Figure 5K; Figure S15, Supporting Information). However, this positive regulatory effect of DRD1 was abrogated by the ATF3 inducer. Similar trends were observed at the mRNA expression of ATF3, CHOP, and associated osteogenic and apoptotic transcripts at the gene level (Figure 5L). Collectively, these results indicate that the upregulation of DRD1 produces positive effects on GC‐induced osteoblastic apoptosis by inhibiting the ATF3/CHOP signaling pathway.

### 2.5 DRD1 Regulates ER Stress and ATF3/CHOP Signaling in a Cyclic AMP‐Dependent Manner

**Figure 6:**
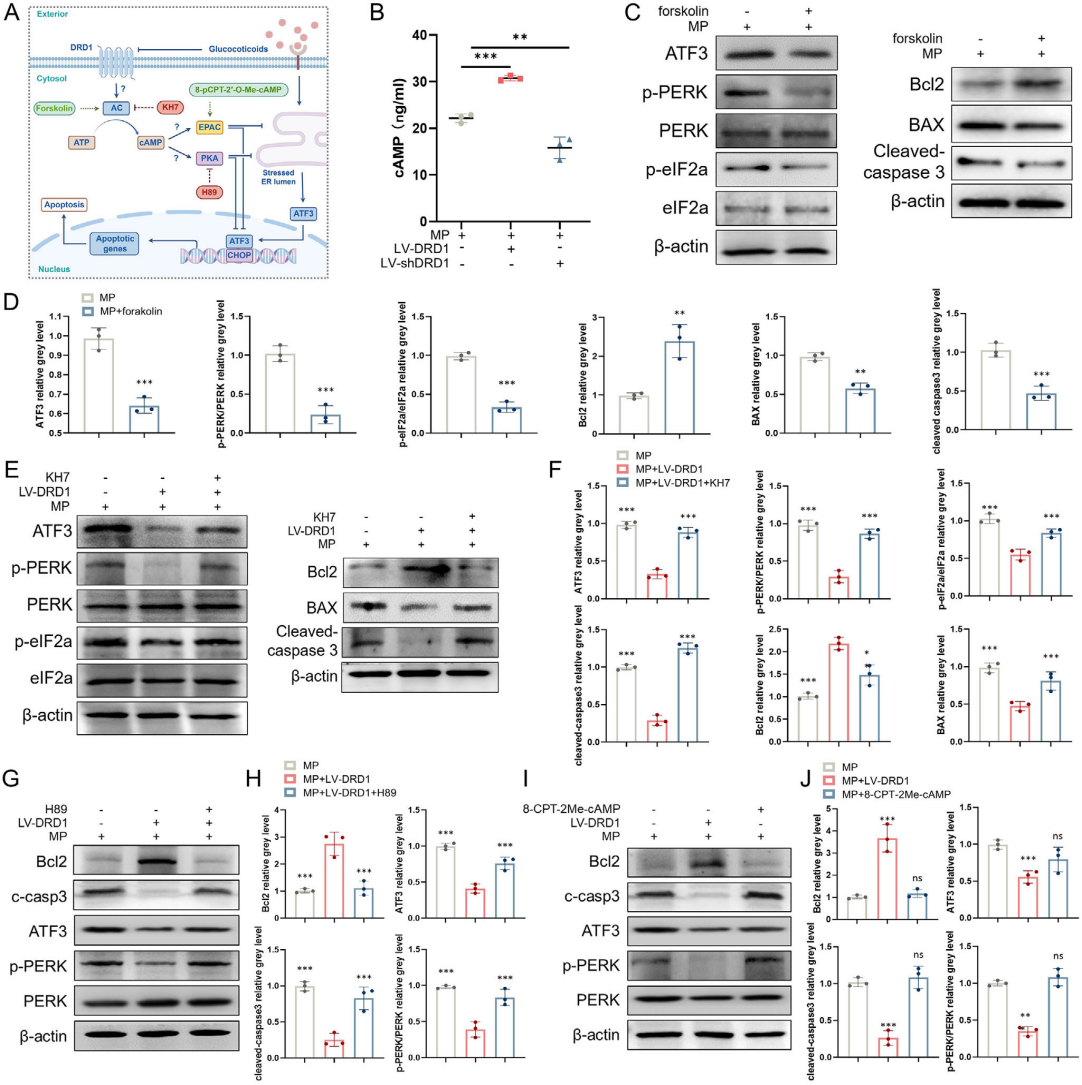
DRD1 regulates ER stress and ATF3/CHOP signaling via cAMP. A) Schematic illustration of the cAMP mechanism explored with related agonists and inhibitors. B) ELISA of cAMP concentration in osteogenesis‐induced BMSCs treated with LV‐DRD1, LV‐shDRD1, and then stimulated with MP. C) Western blotting of ATF3, p‐PERK, PERK, p‐eIF2a, eIF2a, β‐actin, Bcl2, BAX, and cleaved‐caspase3 in osteogenesis‐induced BMSCs treated with forskolin (100 µm) and then stimulated with MP. D) Semiquantitative analysis of these proteins levels. E) Western blotting of ATF3, p‐PERK, PERK, p‐eIF2a, eIF2a, β‐actin, Bcl2, BAX, and cleaved‐caspase3 in osteogenesis‐induced BMSCs treated with KH7 (5 µm), LV‐DRD1 and then stimulated with MP. F) Semiquantitative analysis of ATF3, p‐PERK/PERK, p‐eIF2a/eIF2a, Bcl2, BAX and cleaved‐caspase3 protein levels. G) Western blotting of ATF3, p‐PERK, PERK, β‐actin, Bcl2, and cleaved‐caspase3 in osteogenesis‐induced BMSCs treated with H89 (40 µm), LV‐DRD1 and then stimulated with MP. H) Semiquantitative analysis of ATF3, p‐PERK/PERK, Bcl2, and cleaved‐caspase3 protein levels. I) Western blotting of ATF3, p‐PERK, PERK, β‐actin, Bcl2, and cleaved‐caspase3 in osteogenesis‐induced BMSCs treated with 8‐CPT‐2Me‐cAMP (100 µM), LV‐DRD1 and then stimulated with MP. J) Semiquantitative analysis of ATF3, p‐PERK/PERK, Bcl2 and cleaved‐caspase3 protein levels (*n* = 3 per group, ns denotes not significant, ^**^ denotes *p* < 0.01, ^***^ denotes *p* < 0.001 compared to the MP or MP+LV‐DRD1 group).

Then, we determined how DRD1 exerts its regulatory effect from the cell membrane to ATF3, which is localized in the nucleus. Previous transcriptomic data indicated a significant enrichment of the cAMP signaling pathway following LV‐DRD1 treatment (Figure 5D). As a ubiquitous second messenger, cAMP regulates diverse cellular functions.^[^ ^28^ ^]^ DRD1 is known to activate adenylate cyclase (AC), thereby catalyzing cAMP production.^[^ ^29^ ^]^ Therefore, we investigated the role of cAMP in DRD1‐induced ER stress and apoptosis inhibition (**Figure** **6**A). Under MP conditions, cAMP levels increased in response to LV‐DRD1 and decreased following LV‐shDRD1 treatment (Figure 6B). The AC activator forskolin (a cAMP inducer, 100 µm) effectively inhibited the levels of ATF3, ER stress‐related proteins including phosphorylated protein kinase RNA–like endoplasmic reticulum kinase (PERK) and phosphorylated eukaryotic translation initiation factor 2α (eIF2a), and apoptotic proteins including BAX and cleaved‐caspase 3 (Figure 6C,D). Conversely, DRD1‐induced inhibition of ATF3, ER stress, and apoptosis was specifically blocked by KH7 (an AC inhibitor, 5 µm, Figure 6E,F). These results suggest that DRD1‐induced inhibition of ER stress, ATF3/CHOP signaling, and subsequent osteoblastic apoptosis is cAMP‐dependent.

We further tested which intracellular cAMP sensor was involved in DRD1‐induced inhibition of osteoblastic apoptosis. Protein kinase A (PKA) and exchange protein directly activated by cAMP (EPAC) are two types of cAMP mediators.^[^ ^30^ ^]^ Our results showed that H89 (a PKA inhibitor, 40 µm) blocked the positive effect of LV‐DRD1 on GC‐induced ER stress and apoptosis at the protein level (Figure 6G,H). However, 8‐pCPT‐2′‐O‐Me‐cAMP (an EPAC agonist, 100 µm) had no effect on GC‐induced ER stress or apoptosis (Figure 6I,J). Taken together, these results demonstrate that PKA, but not EPAC, is the effector of cAMP that enhances DRD1‐induced inhibition of ER stress and ATF3/CHOP signaling, ultimately inhibiting osteoblastic apoptosis.

### 2.6 DA Inhibits GC‐Induced Osteoblastic Apoptosis via DRD1

**Figure 7:**
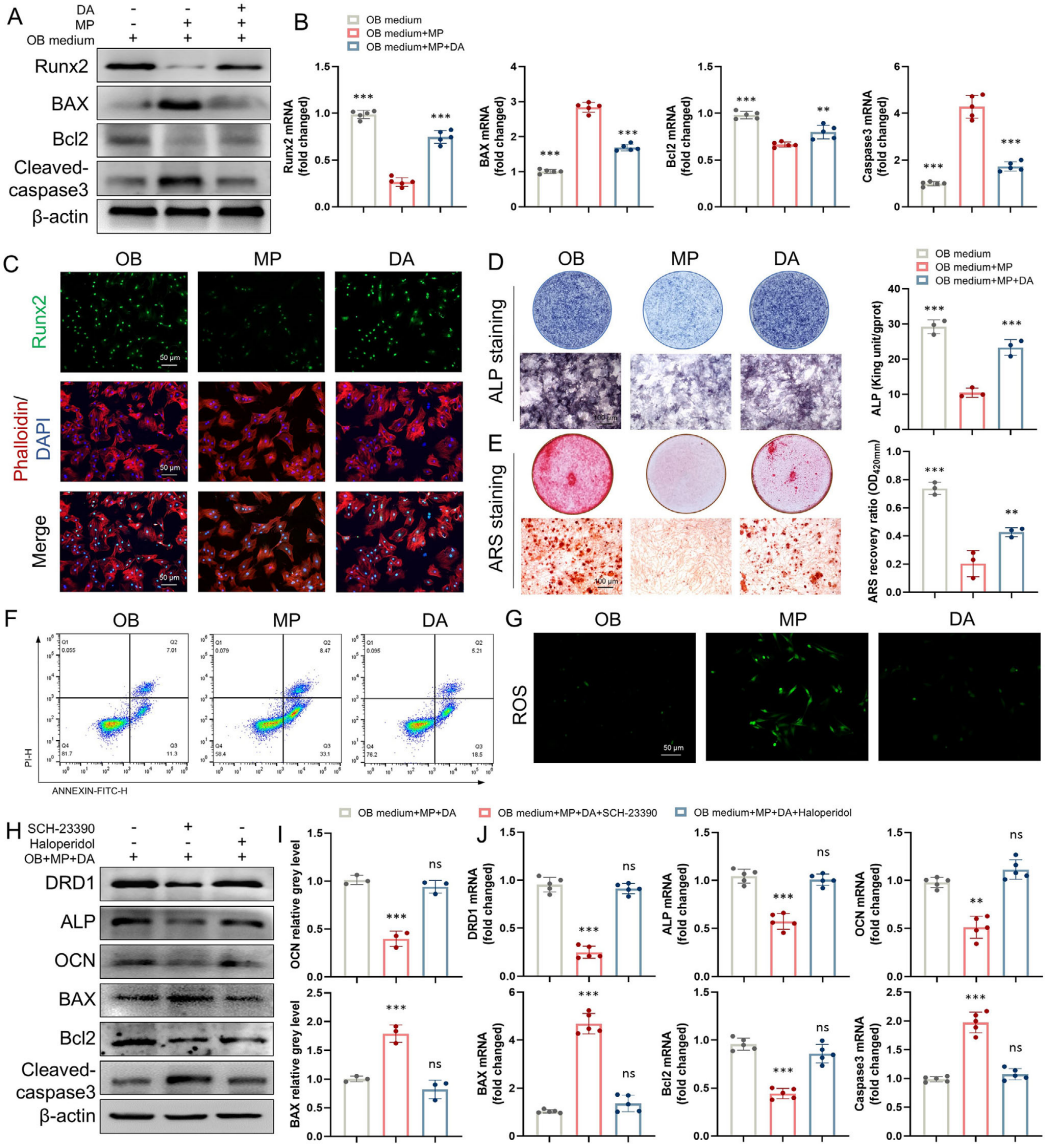
DA inhibits GC‐induced osteoblastic apoptosis via DRD1. A) Western blotting of Runx2, BAX, Bcl2, cleaved‐caspase3, and β‐actin in osteogenesis‐induced BMSCs treated with DA and then stimulated with MP (*n* = 3 per group). B) Gene levels of Runx2, BAX, Bcl2, and Caspase3 in those cells (*n* = 5 per group). C) Immunofluorescence staining of Runx2 (green) in those cells. D) ALP staining of those cells and quantitative evaluation of ALP activity (*n* = 3 per group). E) Alizarin red S staining of those cells and semiquantitative evaluation of ECM mineralization (*n* = 3 per group). F) Flow cytometric analysis of apoptosis in those cells. G) ROS staining of those cells. H) Western blotting of DRD1, ALP, OCN, BAX, Bcl2, cleaved‐caspase3, and β‐actin in osteogenesis‐induced BMSCs treated with SCH‐23390 (1 µm), Haloperidol (1 µm), DA and then stimulated with MP (*n* = 3 per group). I) Semiquantitative analysis of the BAX and OCN protein levels. J) Gene levels of DRD1, ALP, OCN, BAX, Bcl2, and Caspase3 in those cells (*n* = 5 per group, ns denotes not significant, ^**^ denotes *p* < 0.01, ^***^ denotes *p* < 0.001 compared to the OB medium + MP or OB medium + MP + DA group).

DA, an established endogenous agonist of dopamine receptors, was investigated for its potential regulatory effects and mechanisms under GC conditions. The CCK8 assay and inhibition rate of DA showed that no significant cytotoxicity was detected from DA at concentrations below 10 µm after 7 days (Figure S16, Supporting Information). We further evaluated DA's effects on GC‐induced osteogenic inhibition and apoptosis at both protein and gene levels. The levels of the key osteogenic protein Runx2 and the antiapoptotic protein Bcl2 were significantly upregulated following DA treatment. Conversely, the levels of apoptotic proteins including BAX and cleaved‐caspase 3 were markedly down‐regulated (**Figure** **7**A; Figure S17, Supporting Information). The mRNA expression of these characteristic genes also revealed similar patterns (Figure 7B). In addition, the intensity of Runx2 fluorescence in the nucleus was increased by DA under MP intervention (Figure 7C). DA also enhanced ALP activity and mineralization capacity in osteoblasts treated with MP (Figure 7D,E). Flow cytometric analysis provided compelling evidence that DA attenuates MP‐induced osteoblastic apoptosis, with a particularly notable reduction in early apoptotic cells from 33.1% to 18.5% (Figure 7F). Moreover, DA treatment significantly suppressed ROS accumulation in osteoblasts (Figure 7G). These findings demonstrate that DA effectively attenuates GC‐induced osteogenic inhibition and osteoblastic apoptosis.

We further investigated which DA receptor subtype mediates DA‐induced inhibition of osteoblastic apoptosis. DRD1 and DRD2 were downregulated by the specific inhibitors SCH‐23390 and Haloperidol in BMSCs, respectively. The optimal concentration of SCH‐23390 and Haloperidol was chosen as 1 µm based on the CCK‐8 assay and inhibition rate (Figure S18, Supporting Information). Downregulation of DRD1 in BMSCs effectively inhibited the positive regulatory effect of DA on osteogenic inhibition and apoptosis induced by MP, while downregulation of DRD2 had no effect (Figure 7H,I; Figure S19, Supporting Information). Consistently, the mRNA expression of these characteristic osteogenic and apoptotic transcripts was verified at the gene level (Figure 7J). These results suggest that DA suppresses GC‐induced osteoblastic apoptosis primarily via DRD1 activation.

**Figure 8:**
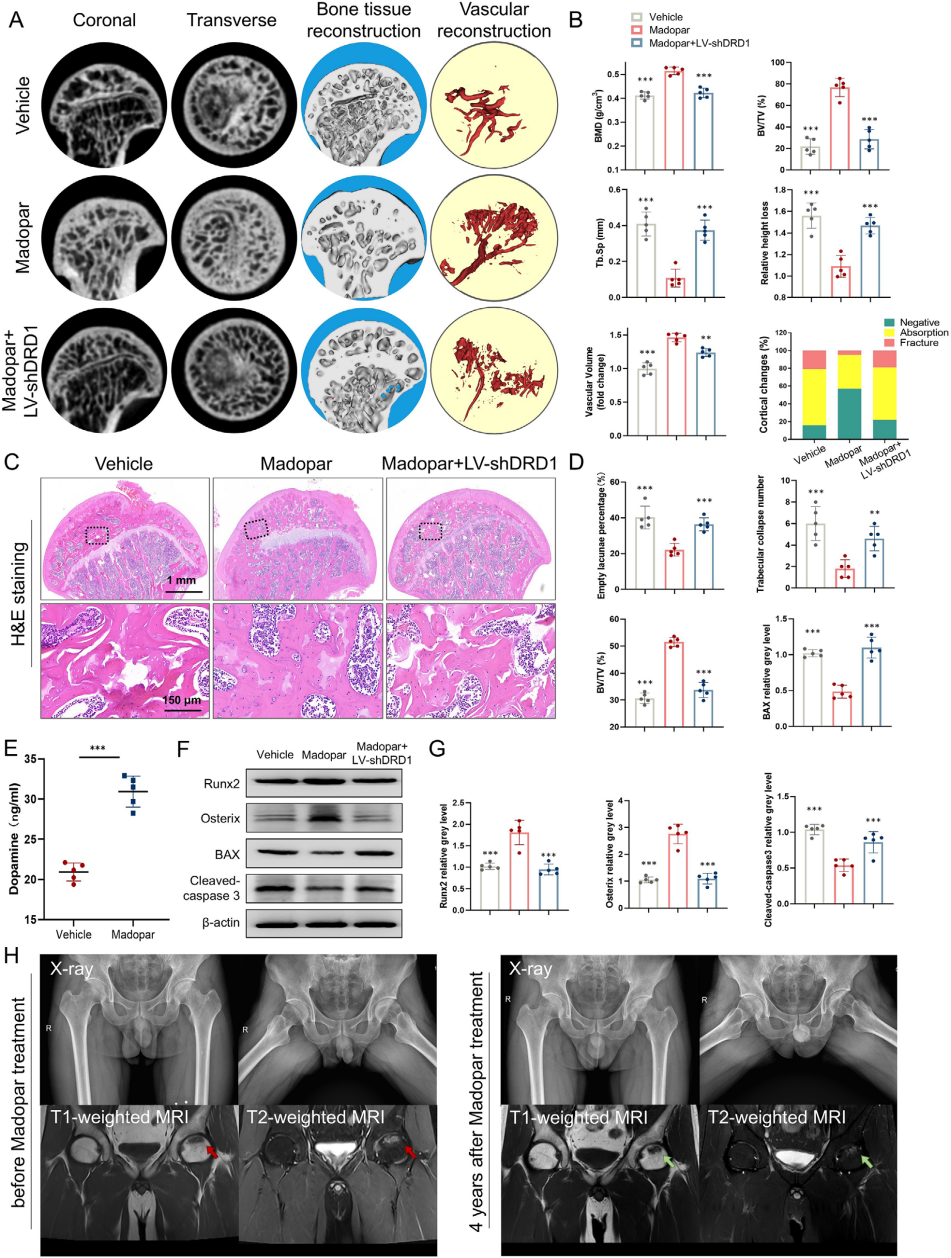
Madopar inhibits GC‐associated ONFH via DRD1. A) Representative images of micro‐CT, 3D reconstruction, and vascular reconstruction of GC‐associated ONFH in coronal, transverse, and profile view. B). BMD (g mm^−3^), BV/TV (%), Tb.Sp (mm), relative height loss, vascular volume, and cortical changes. C) Representative images of H&E staining. D) Empty lacunae percentage (%), trabecular collapse number, and BV/TV (%). E) ELISA of the DA concentration in rat serum. F) Western blot of Runx2, Osterix, BAX, cleaved‐caspase 3, and β‐actin in different tissue samples. G) Semiquantitative analysis of the protein levels (*n* = 5 per group, ^*^ denotes *p* < 0.05, ^**^ denotes *p* < 0.01, and ^***^ denotes *p* < 0.001 compared to the Madopar group). H) Typical case of a GC‐associated ONFH patient before and 4 years after Madopar therapy (red arrows indicate initial ONFH lesion, green arrows indicate the range of ONFH lesion is limited with no collapse and no evidence of progression).

Madopar is an FDA‐approved dopaminergic medication that acts to elevate DA levels.^[^ ^31^ ^]^ It is composed of levodopa and benserazide at a 4:1 ratio. Levodopa crosses the blood‐brain barrier, enters the brain, and is converted into DA via aromatic amino acid decarboxylase (Figure S20, Supporting Information).^[^ ^32^ ^]^ To assess the therapeutic potential of Madopar in GC‐associated ONFH, we evaluated its effects in the GC‐associated ONFH model. Micro‐CT, 3D reconstruction, and vascular reconstruction revealed that Madopar therapeutic effect on pathological manifestations in the femoral head was nearly abolished when DRD1 was suppressed (**Figure** **8**A). Quantitative analysis showed that BMD, BV/TV, and vascular volume decreased significantly, while Tb. Sp, relative height loss, and cortical abnormalities increased following DRD1 inhibition (Figure 8B). In addition, H&E staining verified the histological changes in GC‐associated ONFH upon the LV‐shDRD1 treatment (Figure 8C). An increase in empty lacunae rate and trabecular collapse number was observed when DRD1 was suppressed (Figure 8D). Additionally, BV/TV measurements were significantly lower in the Madopar + LV‐shDRD1 group compared to the Madopar group. We also examined dopamine levels in the serum of GC‐associated ONFH rats treated with or without Madopar. We found that Madopar can effectively elevate serum DA content (Figure 8E). Tissue proteins from the femoral head were extracted and measured by western blot. Our results revealed that inhibition of DRD1 effectively reduced the positive regulatory effect of Madopar on GC‐induced osteogenic inhibition and apoptosis at the protein level (Figure 8F,G). Taken together, these results suggest that Madopar and DA signal through DRD1 to attenuate GC‐induced osteogenic inhibition and apoptosis.

To further study the protective effect of Madopar on GC‐associated ONFH, previous clinical data of GC‐associated ONFH patients treated with this drug at our hospital were retrospectively analyzed. The demographic data in our retrospective study were comparable between the Madopar and non‐Madopar groups (Table S2, Supporting Information). The dimensions of the shadow in the T1‐weighted MRI in a typical case was 3.0 × 1.6 × 1.2cm^3^ before and 2.8 × 1.8 × 1.2 cm^3^ after 4 years Madopar treatment, which indicates no evidence of ONFH progression (Figure 8H). The differences in radiographic and clinical outcomes were also compared between the two groups. At the last follow‐up, the Ficat stage in both groups was exacerbated than pre‐therapy. However, there was no statistically significant difference in the change in the Ficat stage between pre‐therapy and last follow‐up in the Madopar group, but a significant change in the Ficat stage was observed in the control group (Table S3, Supporting Information). The survival analysis of radiographic progression rates and total hip arthroplasty (THA) incidence were also compared (Figure S21, Supporting Information). Endpoint event set to Ficat stage reaches III/IV or patient undergoing THA. Patients in the Madopar group have a significantly higher survival rate. These results suggest that Madopar treatment significantly slows the radiographic progression rates of the Ficat stage and collapse in the femoral head. In addition, the modified Merle d’ Aubigne score at the last follow‐up in the Madopar group was significantly higher than that in the non‐Madopar group, especially in Ficat stage II and III (Table S4, Supporting Information). These results verify that Madopar can potently inhibit GC‐associated ONFH clinically.

## 3 Discussion

Increasing evidence suggests bone acts as an endocrine organ regulating whole‐body metabolism while being modulated by neurotransmitters.^[^ ^33^, ^34^ ^]^ DA and its receptors not only execute central neuromodulation functions but also function serve as local regulators in the periphery.^[^ ^35^ ^]^ However, the specific role of DA receptors in GC‐associated ONFH, particularly in osteoblastic apoptosis, remains poorly understood. Here, we demonstrated that DRD1, but not DRD2, is aberrantly expressed in GC‐associated ONFH. Upregulation of DRD1 inhibits GC‐induced osteoblastic apoptosis, with implications for bone homeostasis and disease progression. We also identified the specific mechanism underlying the inhibitory effect of DRD1 on GC‐induced apoptosis. Finally, the link between Madopar (a clinically approved dopaminergic medication) and DRD1 was proven for GC‐associated ONFH treatment. Together, these findings indicate that DRD1 serves as a critical mediator of the crosstalk between the nervous and skeletal systems.

DA is a catecholamine that functions as both a central neurotransmitter in the brain and a local messenger in peripheral tissues such as blood and bone.^[^ ^36^ ^]^ Our data revealed significantly lower serum DA levels in patients with GC‐ONFH compared with healthy controls, indicating that DA and its receptors are strongly associated with GC‐associated ONFH. There are two major types of DA receptors, termed types D1 and D2, and can be detected in bone cells and tissues.^[^ ^37^ ^]^ Here, our results illustrate that DRD1 exerts a primary role, while DRD2 has no role in GC‐associated ONFH. Consistent with this, Zhu et al. reported that among five DA receptor subtypes (D1‐D5), only the expression level of the D1 subtype was inhibited by GC in a dose‐dependent manner.^[^ ^25^ ^]^ Interestingly, DRD1 is highly expressed in our in vivo clinical samples and rat model of GC‐associated ONFH, while is expressed at low levels after GC treatment in vitro. The discrepancy in results between in vitro and in vivo studies may be mainly attributed to the distinct cell type‐specific expression and localization. In vivo study, the protein and RNA were extracted from the entire femoral head tissues, and the levels of DRD1 represent changes within the entire femoral head. An increase in the number of DRD1‐positive cells was observed in GC‐associated ONFH rats compared to the control group, especially in cartilage, but lower expression of DRD1 was observed in bone marrow in GC‐associated ONFH rats. Meanwhile, In vitro study, we use the BMSCs which originate from bone marrow to evaluate the effect of DRD1. Therefore, the decrease of DRD1 may be due to this reason. In addition, microenvironment crosstalk and negative feedback mechanisms may also be responsible for this discrepancy.^[^ ^38^ ^]^ Regardless, abnormal DRD1 expression can be observed either in vivo or in vitro, indicating that DRD1 might be a potential therapeutic target for GC‐associated ONFH treatment.

Apoptosis is a programmed cell death pathway that responds to metabolic and stress status for maintaining intracellular homeostasis.^[^ ^39^ ^]^ Previous reports have shown that apoptosis is closely associated with GC‐induced imbalances in bone homeostasis, including osteoblast proliferation, differentiation, and bone formation,^[^ ^40^ ^]^ which is consistent with our results. Although DRD1 is a well‐characterized neurotransmitter receptor, its role in bone homeostasis has been gradually specified. Activation of DRD1 has been demonstrated to induce osteogenic differentiation by stimulating the phosphorylation of extracellular regulated protein kinases 1/2 (ERK1/2) and thus enhance Runx2 transcriptional activity.^[^ ^41^ ^]^ In the bone metastasis model, the DRD1 agonist exerts an inhibitory effect on osteoclasts by regulating NFATc1 through increasing p‐eIF2a levels.^[^ ^42^ ^]^ In the osteosarcoma model, DRD1 inhibits osteosarcoma cell proliferation by downregulating ERK1/2 and PI3K‐Akt signaling.^[^ ^43^ ^]^ However, the effect of DRD1 on osteoblasts in GC‐associated ONFH has not been reported. Here, we demonstrate that DRD1 upregulation effectively inhibits GC‐induced osteogenic inhibition, ER stress, and apoptosis both in vitro and in vivo, which adds a new dimension to this field.

While our study has identified a DRD1‐mediated nerve‐bone axis as a novel therapeutic target for GC‐induced osteoblastic apoptosis and ONFH, the underlying mechanism requires further elucidation. ATF3 a member of the ATF/CREB transcription factor family, is rapidly activated by DNA damage and stress stimulation.^[^ ^44^ ^]^ Similar to ATF3, CHOP exerts its transcriptional action in the nucleus, thus inducing cell cycle arrest and apoptosis when ER stress occurs.^[^ ^45^ ^]^ Classic intrinsic apoptosis is initiated by members of the Bcl‐2 family and mitochondria‐derived proteins, which is also involved in our study. However, these typical apoptotic genes are the terminal products of the apoptotic cascade. The regulatory mechanism of DRD1 involved in regulating these typical apoptotic genes is poorly understood. Here, we demonstrate that the upregulation of DRD1 produces positive effects on GC‐induced osteoblastic apoptosis by inhibiting the ATF3/CHOP signaling pathway. Given that DRD1 can increase ADCY activity and cAMP production, which subsequent downstream cAMP sensor is activated warrants exploration. To address this issue, our results demonstrate that PKA, but not EPAC, is the mediator of cAMP to enhance DRD1‐induced inhibition of ER stress, ATF3/CHOP signaling, and subsequent osteoblastic apoptosis. Therefore, targeting DRD1 is promising for modulating the apoptotic response to enhance osteoblast differentiation and skeletal repair.

Importantly, Madopar (an FDA‐approved dopaminergic agent) was used in this study based on our clinical experience. Levodopa, the principal component of Madopar, is originally identified as a regulator of growth hormone for facilitating bone formation, thus is employed in the orthopedic field.^[^ ^46^ ^]^ Jones et al. found that levodopa enhances the healing of a rat mandibular defect.^[^ ^47^ ^]^ Costa et al. reported that 0.2 g kg^−1^ d^−1^ of levodopa administration accelerating nonunion fracture healing, as indicated by significantly greater stiffness, ultimate force, and energy to failure.^[^ ^48^ ^]^ Here, we demonstrate that Madopar treatment can improve pathological and histological changes in GC‐associated ONFH, which supports previous findings. Although Madopar has been identified as a regulator of bone homeostasis, the specific mechanisms underlying the regulatory effect of Madopar on osteoblasts under GC conditions remain unclear. Our results show that Madopar can effectively increase the DA content. In addition, DA signals through DRD1 to attenuate GC‐induced osteogenic inhibition and apoptosis both in vivo and in vitro. The link between Madopar and DRD1, at this point, is clearly demonstrated in our study. This has implications for further research in GC‐associated ONFH treatment by boosting DA levels. In addition, the potential off‐target effects of elevated DA levels, such as gastrointestinal reactions, cardiovascular or psychiatric side effects are noteworthy.^[^ ^49^ ^]^ Strategies that can be considered to minimize systemic exposure include dose adjustment, optimization of administration route, a combination of symptomatic management drugs, genetic testing and individualized dosing, long‐term regular monitoring, and patient education.

In addition, the pathogenesis of ONFH involves multifactorial lipid dysregulation, coagulopathy, osteo‐adipogenic imbalance, autophagy, and inflammation.^[^ ^7^ ^]^ And the DRD1‐mediated apoptosis inhibition may synergize with these pathological pathways. DRD1 may co‐mediate the α1‐AR‐signaling thermogenesis via the SERCA2b/RyR2/CKmt pathway in adipocytes to alleviate osteo‐adipogenic imbalance.^[^ ^50^ ^]^ DRD1 activation may also significantly disrupt autophagic flux via the mTOR pathway to yield a synergistic therapeutic effect.^[^ ^51^ ^]^ In addition, DRD1 signaling negatively regulates NLRP3 inflammasome‐dependent inflammation via the E3 ubiquitin ligase MARCH7, which complements the anti‐apoptotic effect of DRD1.^[^ ^29^ ^]^ Single‐target therapy is difficult to fully intervene in the multi‐pathway pathological network of ONFH, combinatorial therapies are imperative.

There are still some limitations of this study. First, lentiviral DRD1 modulation is experimentally robust, but its clinical translation faces challenges, including immunogenicity, off‐target effects, and delivery limitations. Small‐molecule pharmacological agents targeting DRD1, informed by cryo‐EM structural insights and allosteric modulation mechanisms, offer more practical and clinically feasible alternatives to lentiviral approaches. Prioritizing non‐catechol agonists, allosteric modulators, and subtype‐selective designs can overcome current limitations in blood‐brain barrier penetration, selectivity, and pharmacokinetics, accelerating translation for clinical use. In addition, blood vessel formation and osteoclastic resorption are two other key factors in GC‐associated ONFH.^[^ ^52^ ^]^ DRD1 has been shown to inhibit high glucose‐induced apoptosis via upregulating the CSE/H_2_S pathway in vascular endothelial cells.^[^ ^53^ ^]^ DRD1 activation can also suppress the development of bone‐resorbing osteoclasts by elevating the eIF2α phosphorylation in breast cancer and bone metabolism.^[^ ^42^ ^]^ Thus, the potential role of DRD1 in improving vascularization and osteoclastogenesis would be important to investigate in ONFH progression. Finally, besides the ATF3/CHOP axis, other signaling pathways disrupted in GC‐associated ONFH are not negligible and merit further investigation using knockout models.

## 4 Conclusion

In summary, our study identifies a novel mechanism by which DRD1 contributes to the pathogenesis of GC‐associated ONFH through the regulation of osteoblastic apoptosis. We provide the first evidence demonstrating a functional connection between Madopar and DRD1 in the context of GC‐associated ONFH, suggesting that DRD1 is a promising potential therapeutic target for apoptosis‐driven bone disorders and that Madopar serves as a potential restrictor of progressive GC‐associated ONFH. This work provides groundbreaking insights into DRD1's role in GC‐associated ONFH and offers a compelling case for repurposing Madopar.

## 5 Experimental Section

### Clinical Samples

Clinical sample collection was approved by the Ethics Committee of the First Affiliated Hospital of Soochow University (No. 2023–525). Femoral heads were obtained from two groups of patients after total hip arthroplasty (*n* = 6 group^−1^). One group was diagnosed with GC‐associated ONFH, and the other group was diagnosed with a femoral neck fracture. Only the resected femoral heads from the femur were collected, which caused no additional harm to individuals. Blood samples were obtained from GC‐associated ONFH patients in hospital wards and from healthy volunteers (*n* = 20 group^−1^).

### Clinical Data of Patients with GC‐Associated ONFH Treated with Madopar

The data of 30 patients with GC‐associated ONFH treated with Madopar therapy at the First Affiliated Hospital of Soochow University from October 2002 to May 2013 were retrospectively analyzed. Thirty GC‐associated ONFH patients who did not receive Madopar therapy during the same period were assessed as the control group. The inclusion criteria were: 1) patients who were diagnosed with GC‐associated ONFH in accordance with the latest version of the clinical guidelines (age≤60 years old, no sex restriction); 2) cases of GC‐associated ONFH treated with Madopar or no Madopar at least 2 years follow‐up. Patients were excluded if they had the following: 1) alcoholic ONFH, traumatic ONFH, and other non‐steroid induced ONFH; 2) history of tuberculosis and mental illness; 3) cases with incomplete clinical data.

The patients received Madopar at some time after GC treatment. The administration of Madopar was given in gradually increasing doses. The initial dose was 0.25 g d^−1^ for the first week. If no adverse effects, such as dizziness, nausea, or gastrointestinal problems were observed, the dose was increased to 0.5 g d^−1^ for the second week and 0.75 g d^−1^ for the third week and every week thereafter. The period of administration was 9–12 months based on the evaluation of clinical symptoms and radiographic manifestations. The demographic data, radiographic parameters, and functional outcomes were compared between groups. This retrospective study was authorized by the Ethics Committee of the First Affiliated Hospital of Soochow University (No. 2023–526).

### Experimental Animals and Groups

The animal experiments were approved by the Animal Ethics Committee of the First Affiliated Hospital of Soochow University (No. SUDA20221123A02). Twenty‐week‐old male Sprague‐Dawley rats were used to establish the GC‐associated ONFH model.^[^ ^54^ ^]^ Rats were treated with 2 mg kg^−1^ LPS (Sigma–Aldrich, USA) once daily by intraperitoneal injection for the first three days. Then, rats were treated with 40 mg kg^−1^ methylprednisolone (MP, Pfizer, USA) once daily by intramuscular injection in the gluteal muscle for the next six days. MP injections were alternated daily between the two sides of the gluteal muscle. The duration of GC‐associated ONFH induction was 6 weeks after MP medication.^[^ ^15^, ^55^ ^]^

Forty‐nine rats were randomly divided into the following groups: the control group (PBS treatment, *n* = 7), vehicle group 1 (GC‐associated ONFH, *n* = 7), vehicle group 2 [GC‐associated ONFH + lentiviral negative control (LV‐NC) treatment, *n* = 7], Madopar group (GC‐associated ONFH + LV‐NC + Madopar treatment, *n* = 7), DRD1‐overexpressing lentivirus (LV‐DRD1) group (GC‐associated ONFH + LV‐DRD1 treatment, *n* = 7), DRD1‐silencing lentivirus (LV‐shDRD1) group 1 (GC‐associated ONFH + LV‐shDRD1 treatment, *n* = 7) and LV‐shDRD1 group 2 (GC‐associated ONFH + LV‐shDRD1 + Madopar treatment, *n* = 7). The LV‐DRD1 and LV‐shDRD1 groups were treated with 100 µL of LV‐DRD1 and LV‐shDRD1 (5 × 10^8^ PFU mL^−1^, GenePharma, China), respectively, by intramuscular injection on the 2^nd^ day after MP injection. The control group was treated with PBS, and the vehicle group was treated with LV‐NC under the same condition. In addition, Madopar (0.2 g kg^−1^, Roche, USA) was administered on the 2^nd^ day after MP injection daily via oral gavage until the end of the study period. The femoral heads were harvested for further evaluation at 6 weeks after MP medication.

### Cell Culture and Differentiation

BMSCs were isolated from the tibia and femur of Sprague‐Dawley rats and cultivated in α‐MEM supplemented with 10% FBS (Gibco, USA) at 37 °C in 5% CO_2_. For osteogenic differentiation, BMSCs were cultured in an osteogenic medium containing 10 mM β‐glycerophosphate, 0.5 mm ascorbic acid, and 0.1 µm dexamethasone.

### Cell Viability Assay

The cytotoxicity of MP, dopamine (Sigma–Aldrich, USA), SCH‐23390 (MCE, USA), and Haloperidol (MCE, USA) to BMSCs was measured by the CCK‐8 kit assay (Beyotime, China). Cells were seeded in 96‐well plates at 2000 cells well^−1^ with different concentrations of the corresponding drugs and cultured for 1, 4, and 7 days. Next, cells were incubated with 100 µl of culture medium containing 10% CCK‐8 solution. The absorbance at 450 nm was monitored after incubating for 1 h at 37 °C.

### Western Blot Analysis

Proteins were extracted using radio immunoprecipitation assay, then the bicinchoninic acid kit was performed to quantify the concentration. After proteins were separated, the blots were blocked and incubated with primary antibodies, including antibodies against DRD1 (1:1000, ab20066, Abcam, UK), DRD2 (1 µg mL^−1^, ab85367, Abcam, UK), Runx2 (1:1000, A2851, Abclonal, China), Osterix (1:1000, A18699, Abclonal, China), OCN (1:1000, orb1266, Biorbyt, UK), ALP (1:1000, DF4722, Affinity Biosciences, USA), BAX (1:1000, A0207, Abclonal, China), Bcl2 (1:1000, A19693, Abclonal, China), cleaved‐caspase 3 (1:1000, orb126608, Biorbyt, UK), β‐actin (1:10 000, AC026, Abclonal, China), ATF3 (1:1000, abs115809, Absin, China), CHOP (1:2000, A0221, Abclonal, China), p‐PERK (1:1000, AP0886, Abclonal, China), PERK (1:1000, A18196, Abclonal, China), p‐eIF2a (1:2000, AP0692, Abclonal, China), eIF2a (1:500, A0764, Abclonal, China). The corresponding secondary antibodies were then used at 1:5000 (Beyotime, China). Protein bands were detected using a ChemiDoc gel imaging system (Bio‐Rad, USA), and relative gray levels were analyzed by ImageJ (Bethesda, USA).^[^ ^56^ ^]^

### Lentivirus Infection

DRD1 overexpression and knockdown lentivirus were synthesized by GenePharma. Cells were infected by lentivirus accordance with the instruction manual. The infection typically lasted 48–72 h before further experimentation. The infectious efficiency was detected using fluorescence microscopy (Zeiss, Germany) and measured using ImageJ (Bethesda, USA). The regulatory effects of DRD1 were identified at the gene and protein levels.

### Quantitative Real‐Time PCR Assay

Total RNA was extracted using TRIzol reagent and complementary DNA (cDNA) was synthesized from RNA by reverse transcription. Then, cDNA was amplified using a CFX96 real‐time system (Bio‐Rad, USA). Primers for target genes are listed in Table S5 (Supporting Information).

### ELISA Assay

The concentrations of dopamine in culture medium and serum, and cAMP in cell supernatant were tested using ELISA kits (Elabscience, China) according to the manufacturer's instructions. The absorbance at 450 nm was recorded using a spectrophotometer (Bio‐Rad, USA).

### ALP Staining

After treatment, the cells were fixed and incubated with a BCIP/NBT (Beyotime, China) working solution in the dark. The activity of alkaline phosphatase was detected by an ALP quantification kit (Jiancheng Bioengineering Institute, China). The absorbance at 520 nm was monitored using a spectrophotometer (Bio‐Rad, USA).

### Alizarin Red S Staining

After treatment, the cells were fixed and incubated with 0.1% ARS staining solution (pH 4.1, Beyotime, China) in the dark. The stained calcium nodules were captured microscopically. Then, the calcium nodules were dissolved in a 5% perchloric acid solution, and the absorbance at 420 nm was monitored using a spectrophotometer (Bio‐Rad, USA).

### Flow Cytometric Analysis

The cells were collected after different treatments and double‐stained with Annexin V‐FITC and propidium iodide (PI, Yeasen, China). Then, the binding buffer was added and samples were determined by flow cytometry (Merck Millipore, Germany). The quadrant positioning of Annexin/PI plots was conducted to distinguish living (Annexin V‐/PI‐), early apoptotic (Annexin V+/PI‐), late apoptotic (Annexin V+/PI+), and necrotic (Annexin V‐/PI+) cells using FlowJo (BD Biosciences, USA).

### Transmission Electron Microscopy

After different treatments, the cells were fixed with 2.5% glutaraldehyde at 4 °C overnight, followed by fixation in 1% osmium acid for 2 h. Then, the samples were embedded, ultrathin sectioned, and stained with uranyl acetate and lead citrate. TEM images were captured using an H‐7800 TEM (Hitachi, Japan) at 80 kV.

### Cell Immunofluorescence Staining

After different treatments, the cells were fixed and permeabilized. Then, the cells were incubated with primary antibodies against OCN (1:100, orb1266, Biorbyt, UK), Runx2 (1:200, A2851, Abclonal, China), and ATF3 (3 µg mL^−1^, ab191513, Abcam, UK). The corresponding secondary fluorescent antibody was then used at 1:1000 (Alexa Fluor 488, Abcam, UK). Phalloidin (Yeasen, China) and DAPI (Beyotime, China) were used to stain the cytoskeleton and nuclei. Fluorescence images were acquired using a fluorescence microscope (Zeiss, Germany).

### ROS Staining

The cells after different treatments were dark‐incubated with DCFH‐DA working solution (1:1000 dilution) and rinsed with α‐MEM. The ROS‐stained cells were captured using a fluorescence microscope (Zeiss, Germany).

### RNA Sequencing and Bioinformatics

After different treatments, the cells were harvested and the total RNA was isolated. The concentration was quantified using an Agilent 2100 Bioanalyzer (Agilent Technologies, USA). Transcriptome sequencing was performed by Gene Denovo Biotechnology (China). Clean reads of RNA‐seq were mapped onto the reference genome using HISAT2 and FPKM values were calculated using Cufflinks. Differential expression of transcripts was identified by KEGG, GO, and gene set enrichment analysis. Genes with an FDR less than 0.05 and fold change greater than 2 were considered as statistically differentially expressed transcripts. The hierarchical clustering analysis was depicted in a heat map format.

### Histological, Immunohistochemical, and Colocalization Staining

The collected femoral heads were fixed and decalcified with 10% EDTA for 4 weeks. Samples were then embedded in paraffin and sectioned into 6 µm slices. Routine H&E staining was used to evaluate the morphological changes of the specimens. The empty lacunae rate, trabecular collapse number, and BV/TV in femoral heads were determined. IHC staining was performed to determine the expression of DRD1. The number of DRD1‐positive cells in the region of interest was quantified. Colocalization staining was performed to confirm DRD1 expression in osteoblasts through immunofluorescence staining. Images of the stained sections were acquired using an Axio‐Cam HRC or fluorescence microscope (Zeiss, Germany).

### Micro‐CT Analysis

Radiographic changes were evaluated using a high‐resolution micro‐CT SkyScan1176 system (Bruker, Belgium). The scanner was set at a voltage of 65 kV, current of 385 µA, and resolution of 18 µm per pixel. 3D image reconstruction was then performed, and the quantitative parameters of the bone microstructure, including the BMD, BV/TV, Tb.N, Tb.Th, Tb.Sp and relative height loss were measured. Cortical changes in the femoral head were also assessed.

### Angiography

The rats were anesthetized and fixed on the operating table. The abdominal aorta was exposed and an arterial catheter was prepared for vascular irrigation and fixation using heparin saline and formalin under appropriate pressure. Then, 20 mL of Microfil (Flow Tech, USA) was slowly injected into each rat at a uniform rate, and rats were sacrificed during this process. The femoral heads were harvested after 12 h storage at 4 °C for complete contrast agent polymerization. The decalcified femoral head was then scanned and analyzed using micro‐CT as described above, and a 3D model of the femoral head angiography was created.

### Calcein‐Alizarin Red S Labeling

Rats were double stained by intraperitoneal injection of 10 mg kg^−1^ calcein and 30 mg kg^−1^ alizarin red (Sigma–Aldrich, USA) at 10 and 3 days before sacrifice. Undecalcified samples were fixed, dehydrated, and then sectioned. The parameters, including MS/BS and MAR, were measured. The fluorescent labeling images were acquired using a fluorescence microscope (Zeiss, Germany).

### TUNEL Assay

In vivo apoptosis was evaluated by the TdT‐mediated dUTP nick‐end labeling (TUNEL, Beyotime, China) assay according to the manufacturer's protocol. The number of TUNEL‐positive cells in the region of interest was quantified.

### Statistical Analysis

Data were represented as means ± standard deviation. The Student's t‐test was used to verify the significance of differences between the two groups. The one‐way ANOVA analysis and Dunnett's multiple comparisons test were performed for multiple comparisons. The Kaplan–Meier analysis was used to assess survivorship. The statistical analysis was performed using GraphPad Prism 8.0 software. Statistical significance was defined as *p* < 0.05.

## Conflict of Interest

The authors declare no conflict of interest.

## Supporting information



Supporting Information

## Data Availability

The data that support the findings of this study are available in the supplementary material of this article.
