# Supplementary material for: Dopamine D1 Receptor Contributes to Glucocorticoid‐Associated Osteonecrosis of Femoral Head Protection Through the ATF3/CHOP Axis to Inhibit Osteoblastic Apoptosis
Source: Adv Sci (Weinh). 2025 Jun 29;12(33):e02276. doi: 10.1002/advs.202502276 (PMC12412510; doi:10.1002/advs.202502276)
Supplement: Supplementary file 1 — Supporting Information [file ADVS-12-e02276-s001.docx]

Supporting Information

Dopamine D1 receptor contributes to glucocorticoid-associated osteonecrosis of femoral head protection through the ATF3/CHOP axis to inhibit osteoblastic apoptosis

Kai Zheng, Wenming Li, Tianhao Wang, Gaoran Ge, Wei Zhang, Yi Qin, Wenhao Li, Zebin Wu, Zhen Wang*, Gang Rui*, Yaozeng Xu*, Dechun Geng*

K. Zheng, W. Li, G. Ge, W. Zhang, Y. Qin, W. Li, Z. Wu, Y. Xu, D. Geng

Department of Orthopedics, The First Affiliated Hospital of Soochow University, Suzhou, China

E-mail: xuyaozeng@suda.edu.cn (Y. Xu); szgengdc@suda.edu.cn (D. Geng)

K. Zheng, G. Rui

Department of Orthopedics, The First Affiliated Hospital of Xiamen University, School of Medicine, Xiamen University, Xiamen, China

E-mail: ruigang@xmu.edu.cn (G. Rui)

Z. Wang

Department of Orthopedics, Suzhou Kowloon Hospital Shanghai Jiao Tong University School of Medicine, Suzhou, Jiangsu, China

E-mail: bigzhen0512@alu.suda.edu.cn (Z. Wang)

T. Wang

Department of Orthopedics, Wuxi Ninth People's Hospital Affiliated to Soochow University, Wuxi, China

K. Zheng, W. Li, T. Wang contributed equally to this work

| **Table S1. GC-associated ONFH patients’ information** | | | |
| --- | --- | --- | --- |
| Patients | Diagnosis | Dose | Treatment duration |
| No.1 | Systemic lupus erythematosus | Pulse therapy of 800 mg/day for 3 days, and maintenance therapy of 0.5 mg/kg/day | 6 months |
| No.2 | Myasthenia gravis | Prednisone 0.5mg per kilogram of body weight is gradually increased by 5 mg every 3 days until the full dose (60 mg) is achieved | 5 months |
| No.3 | Rheumatoid arthritis | Prednisone 40 mg daily can be used. After symptom control, it was gradually reduced and maintained at 10 mg daily. And gradually use of non-steroidal anti-inflammatory drugs for replacement | According to the symptoms |
| No.4 | Systemic lupus erythematosus | Pulse therapy of 700 mg/day for 3 days, and maintenance therapy of 0.5 mg/kg/day | 5 months |
| No.5 | Multiple sclerosis | Starting at 1 g/day for 5 days, and then the dose ladder is halved to 2 days per dose. When the dose was less than 120 mg, it was changed to 60 mg orally, once a day. Each dose was reduced for 2 days, and then gradually halved until the dose was stopped | 1 month |
| No.6 | Nephrotic syndrome | Prednisone 1 mg/kg/d was given at full dose for 8 weeks, decreasing by 5 mg every 2 weeks to maintain at a minimum effective dose of 15 mg | 1.5 years |

**
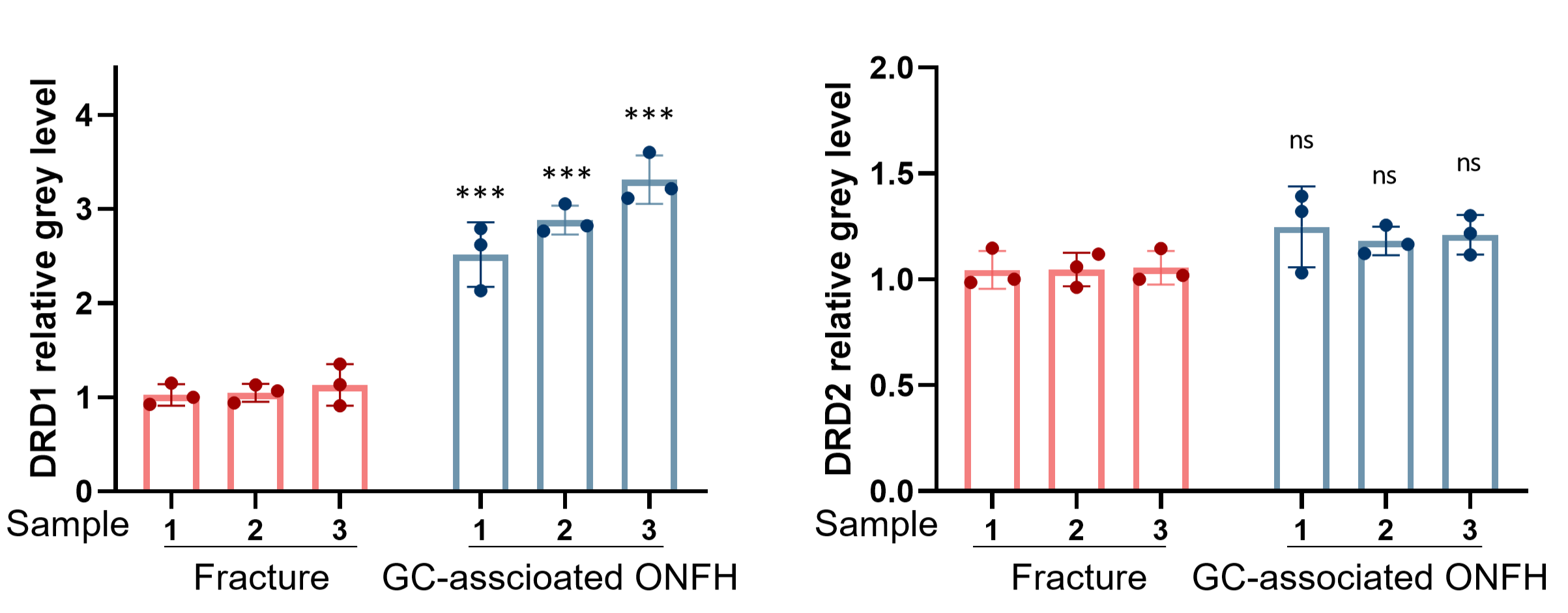
**

**Figure S1.** Semiquantitative analysis of the DRD1 and DRD2 protein levels in clinical samples (n=3 per group, ns denotes not signiﬁcant, *** denotes *p* < 0.001 compared to the fracture group).


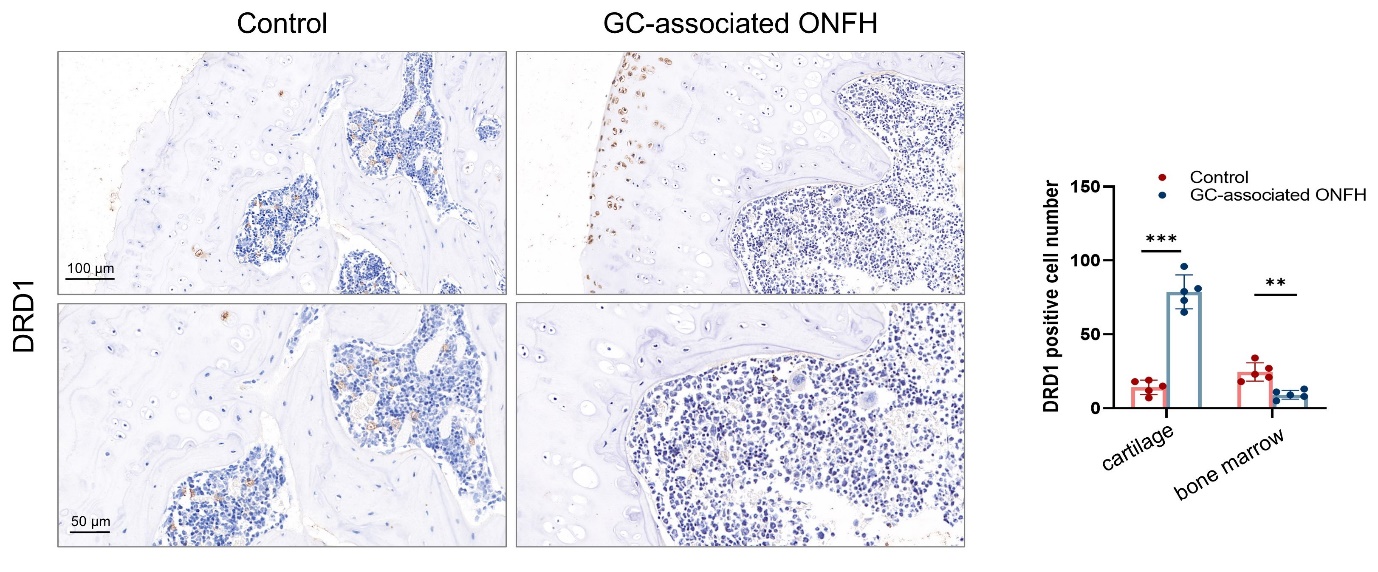


**Figure S2.** IHC staining and quantitative analysis of DRD1 in control and GC-associated ONFH rats (n=5 per group, ** denotes *p* < 0.01, *** denotes *p* < 0.001 compared to the control group).

**
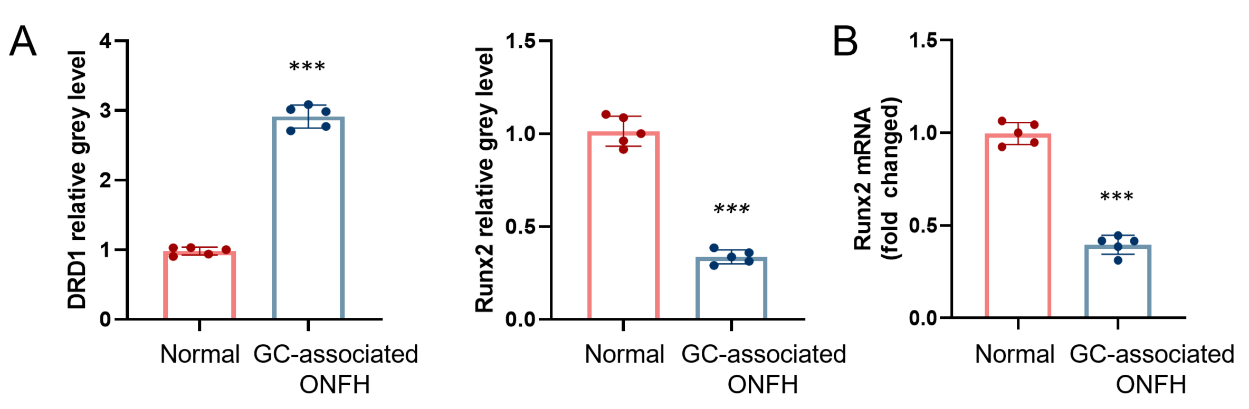
**

**Figure S3.** A) Semiquantitative analysis of the DRD1 and Runx2 protein levels in normal and GC-associated ONFH rats. B) Gene levels of Runx2 (n=5 per group, *** denotes *p* < 0.001 compared to the normal group).

**
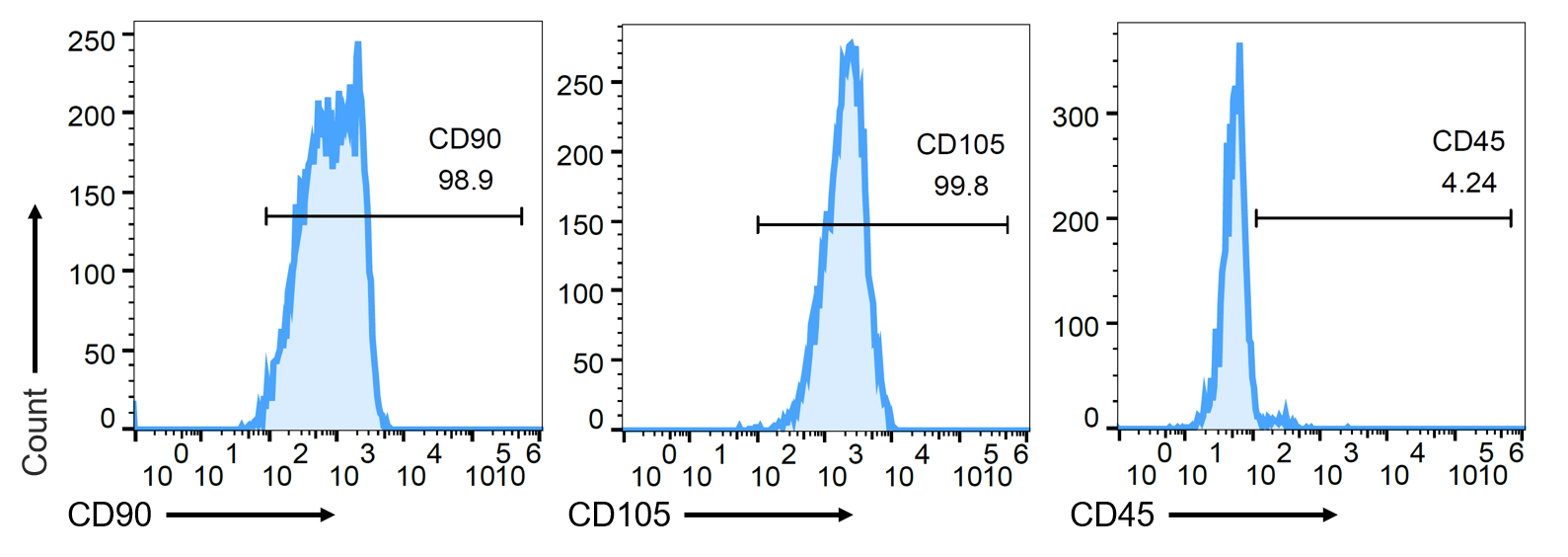
**

**Figure S4.** Isolation and identification of BMSCs by flow cytometry.


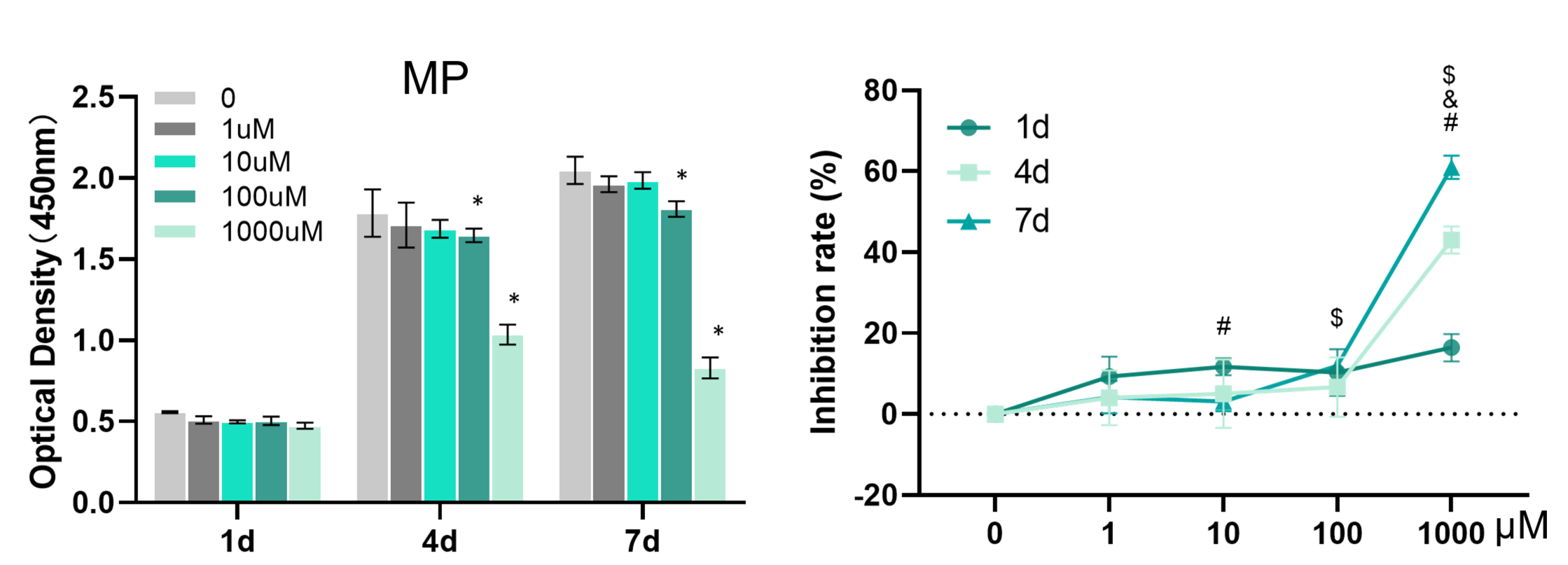


**Figure S5.** Viability and inhibition rate of the BMSCs after incubation with MP at different concentrations (0-1000 µM) for 1, 4 and 7d (n=5 per group, *, ^#^(1d), ^&^(4d), ^$^(7d) denotes *p* < 0.05 compared to the basal group).


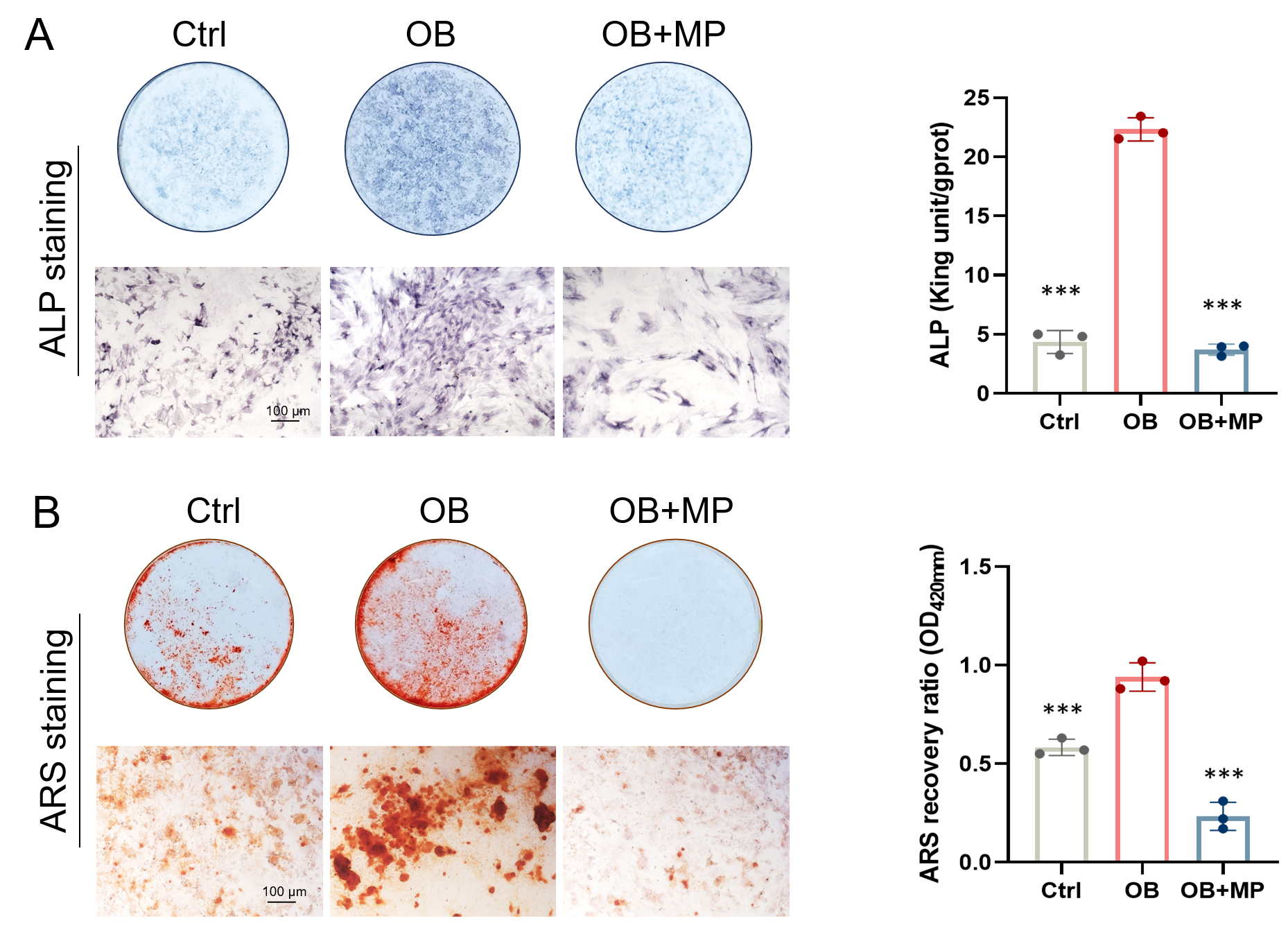


**Figure S6.** A) ALP staining of BMSCs, osteogenesis-induced BMSCs and MP-stimulated osteogenesis-induced BMSCs and quantitative evaluation of ALP activities. B) Alizarin red S staining of those cells and semiquantitative evaluation of ECM mineralization (n=3 per group, *** denotes *p* < 0.001 compared to the osteogenic induction group).


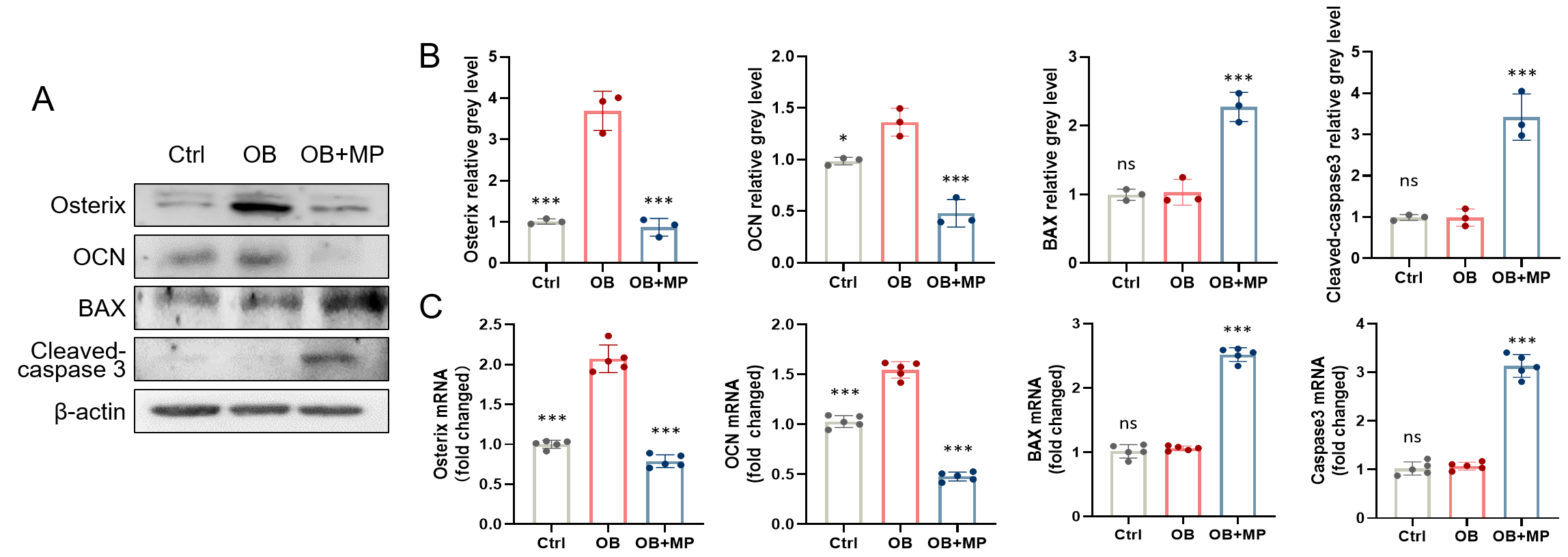


**Figure S7.** A) Western blot of Osterix, OCN, BAX, cleaved-caspase 3 and β-actin in BMSCs, osteogenesis-induced BMSCs and MP-stimulated osteogenesis-induced BMSCs (n=3 per group). B) Semiquantitative analysis of the protein levels. C) Gene levels of Osterix, OCN, BAX and caspase 3 in those cells (n=5 per group, ns denotes not signiﬁcant, * denotes *p* < 0.05, *** denotes *p* < 0.001 compared to the osteogenic induction group).


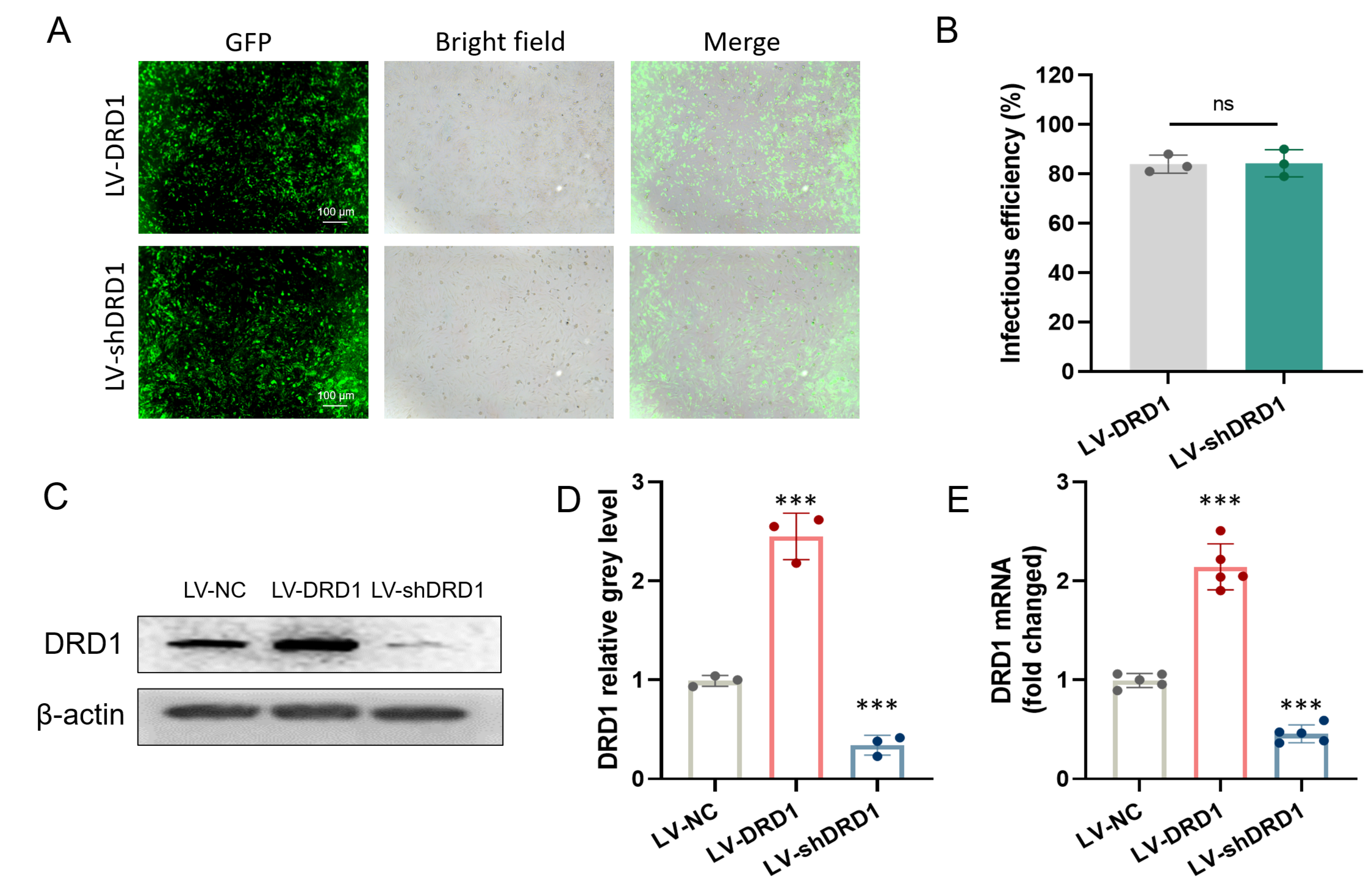


**Figure S8.** A) Representative images of bright field and GFP fluorescence photographs in BMSCs with LV-DRD1 and LV-shDRD1. B) Semiquantitative evaluation of infectious efficiency (n=3 per group). C) Western blot of the DRD1 in BMSCs after lentivirus infection (n=3 per group). D) Semiquantitative analysis of the DRD1. E) Gene levels of DRD1 (n=5 per group, ns denotes not signiﬁcant, *** denotes *p* < 0.001 compared to the LV-NC group).


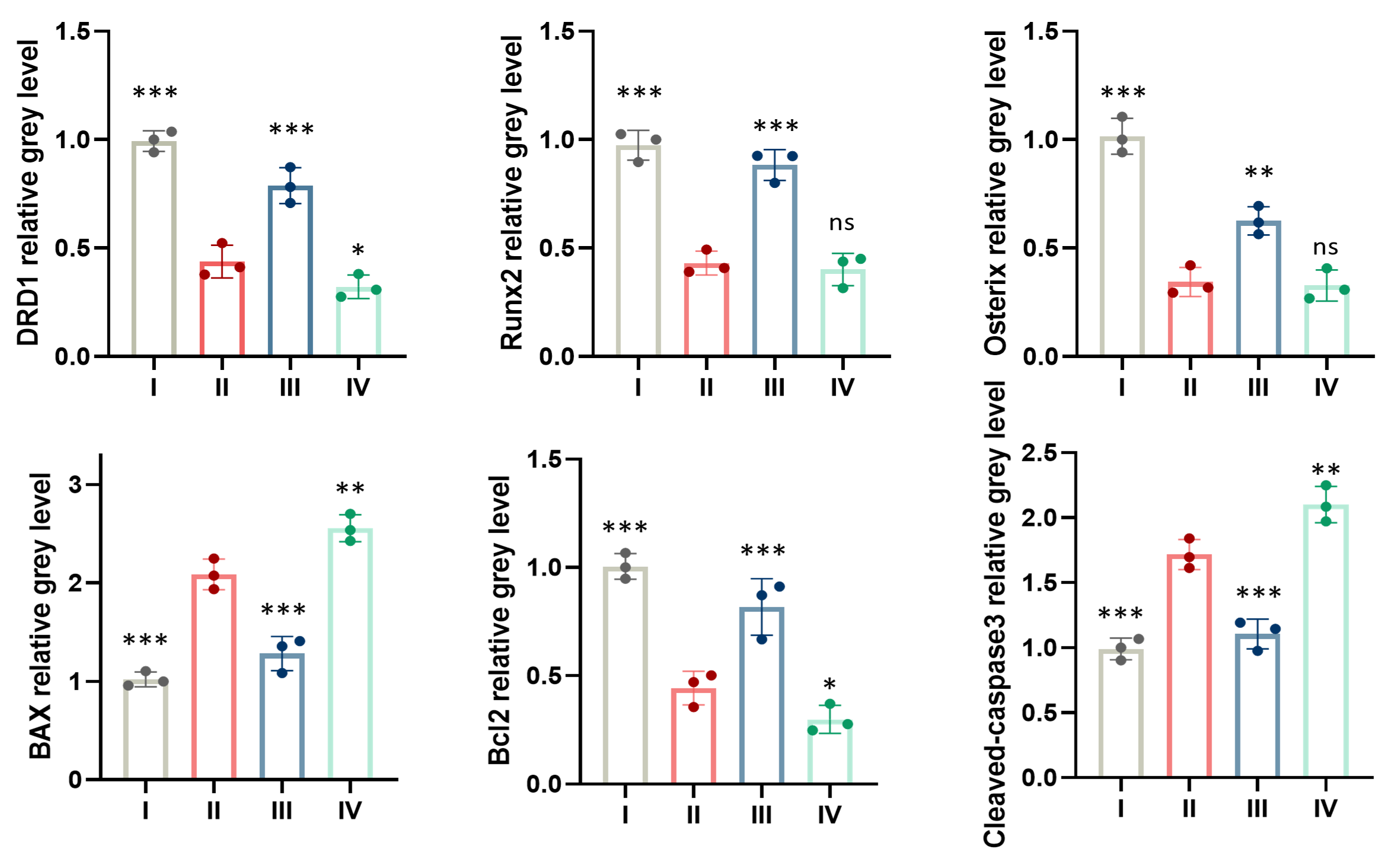


**Figure S9.** Semiquantitative analysis of the DRD1, Runx2, Osterix, BAX, Bcl2 and cleaved-caspase3 protein levels. (I: LV-NC + Osteogenic induction, II: LV-NC + Osteogenic induction + MP, III: LV-DRD1 + Osteogenic induction + MP, IV: LV-shDRD1 + Osteogenic induction + MP, n=3 per group, ns denotes not signiﬁcant, * denotes *p* < 0.05, ** denotes *p* < 0.01, and *** denotes *p* < 0.001 compared to the LV-NC + Osteogenic induction + MP group).


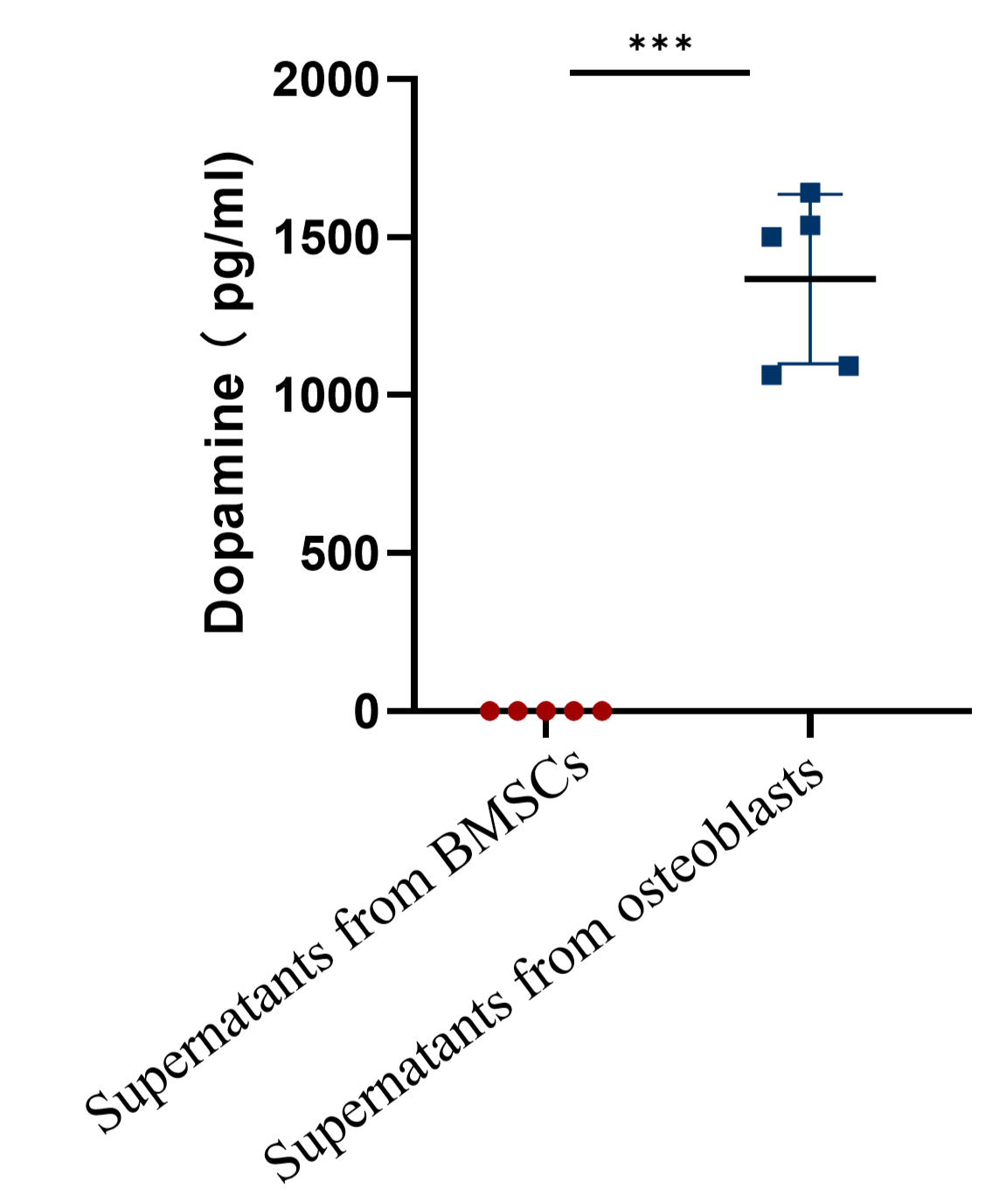


**Figure S10**. ELISA of the DA concentration in culture medium supernatants from BMSCs and osteogenic culture medium supernatants from osteoblasts after 3 days incubation (n=5 per group, *** denotes *p* < 0.001).

**
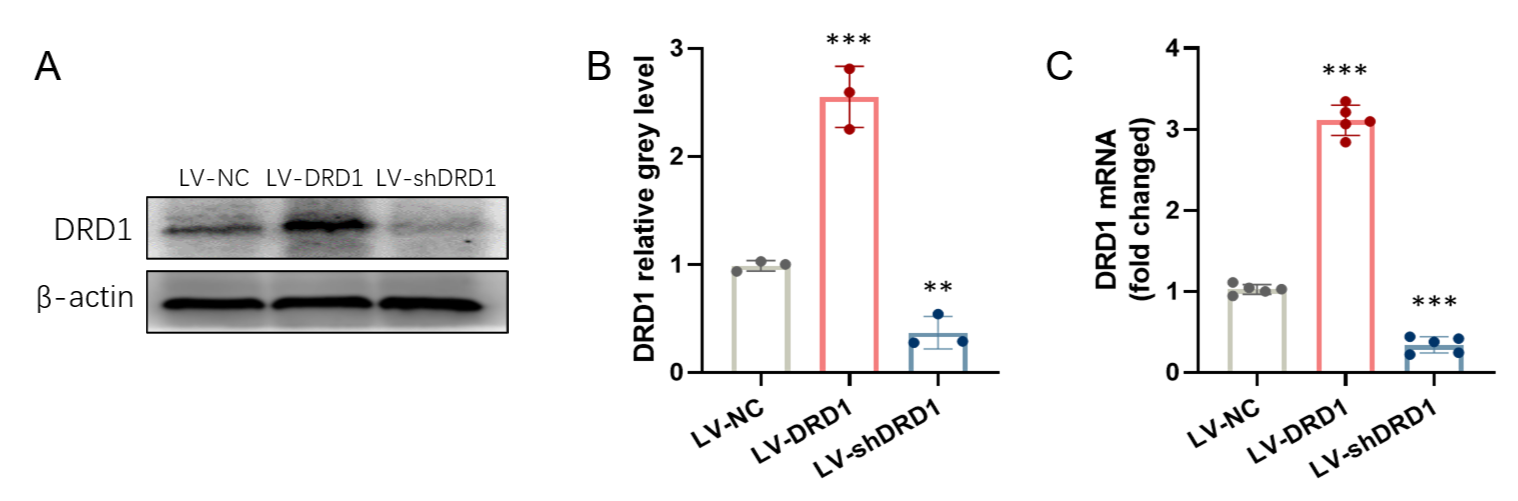
**

**Figure S11.** A) Western blot of the DRD1 in different GC-associated ONFH rat samples after lentivirus infection (n=3 per group). B) Semiquantitative analysis of the DRD1. C) Gene levels of DRD1 (n=5 per group, ** denotes *p* < 0.01, *** denotes *p* < 0.001 compared to the LV-NC group).

**
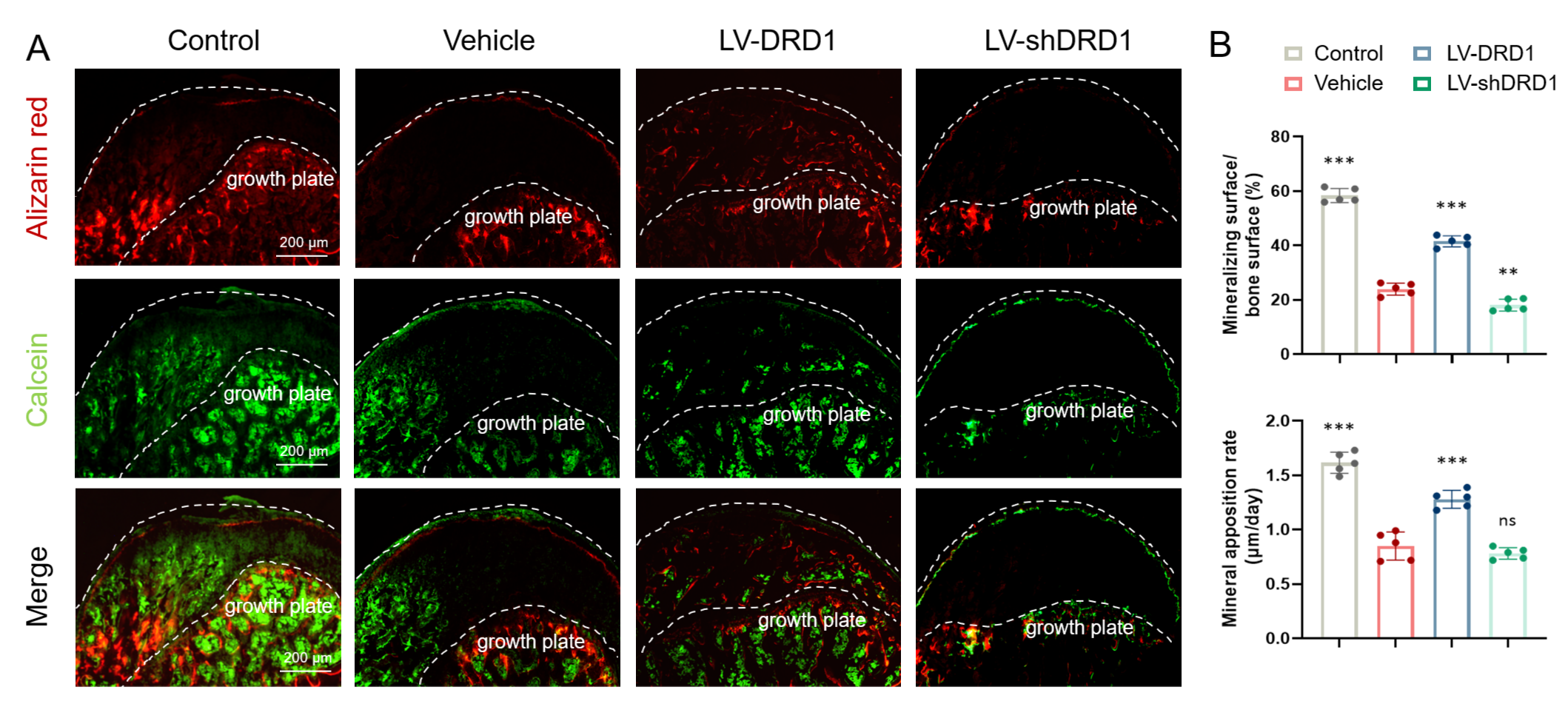
**

**Figure S12.** A) Representative images of calcein-Alizarin red S double labeling. B) Mineralizing surface/bone surface (%), mineral apposition rate (μm/day, n=5 per group, ns denotes not signiﬁcant, ** denotes *p* < 0.01, *** denotes *p* < 0.001 compared to the vehicle group).


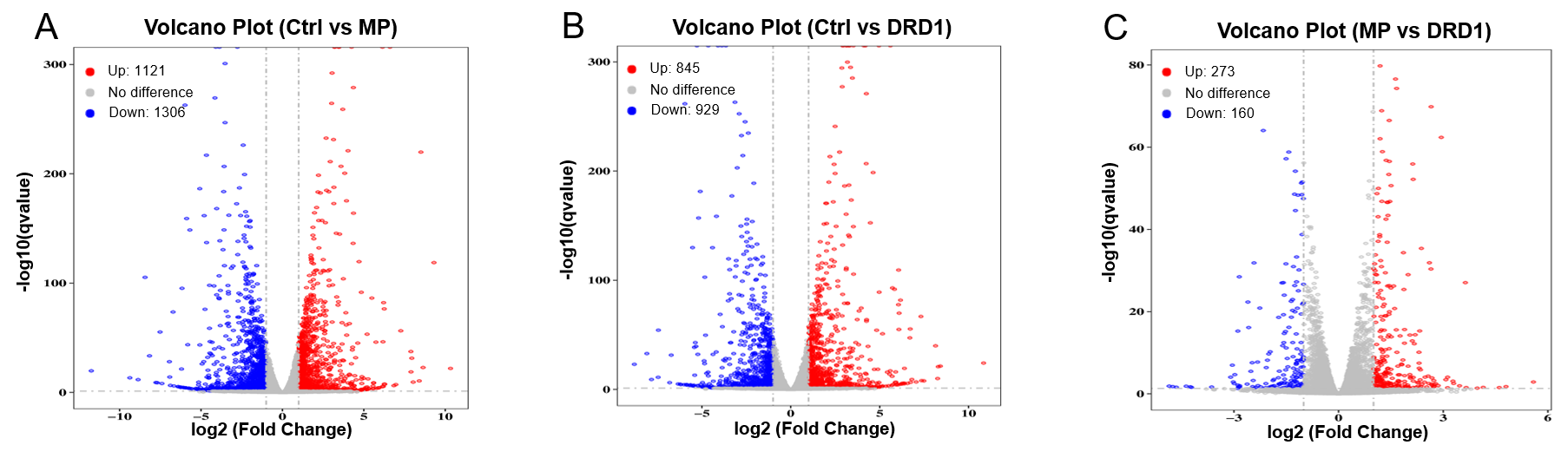


**Figure S13.** A) Volcano map of DEGs in the MP group compared to the control group. B) Volcano map of DEGs in the MP+LV-DRD1 group compared to the control group. C) Volcano map of DEGs in the MP+LV-DRD1 group compared to the MP group.


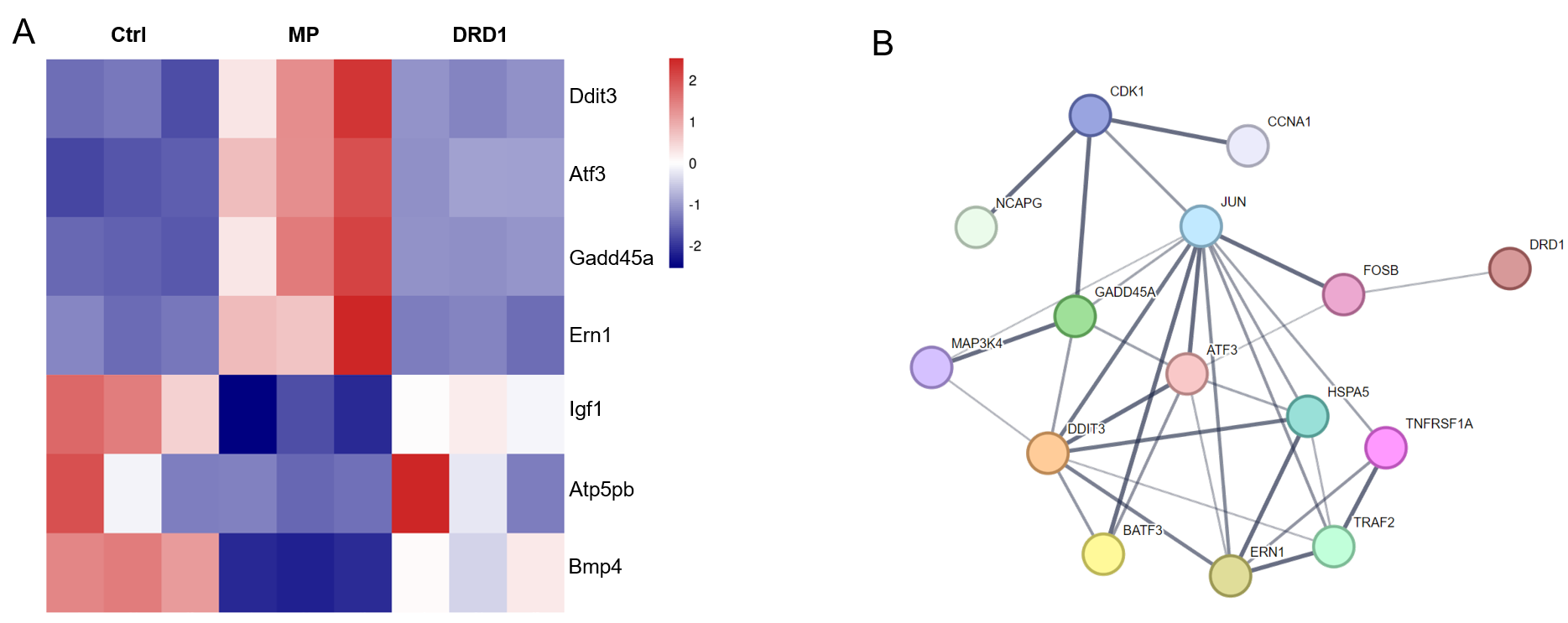


**Figure S14.** A) Local heat map of DEGs. B) STRING analysis showed the functional protein-protein interaction networks of DRD1 and the aforementioned genes.


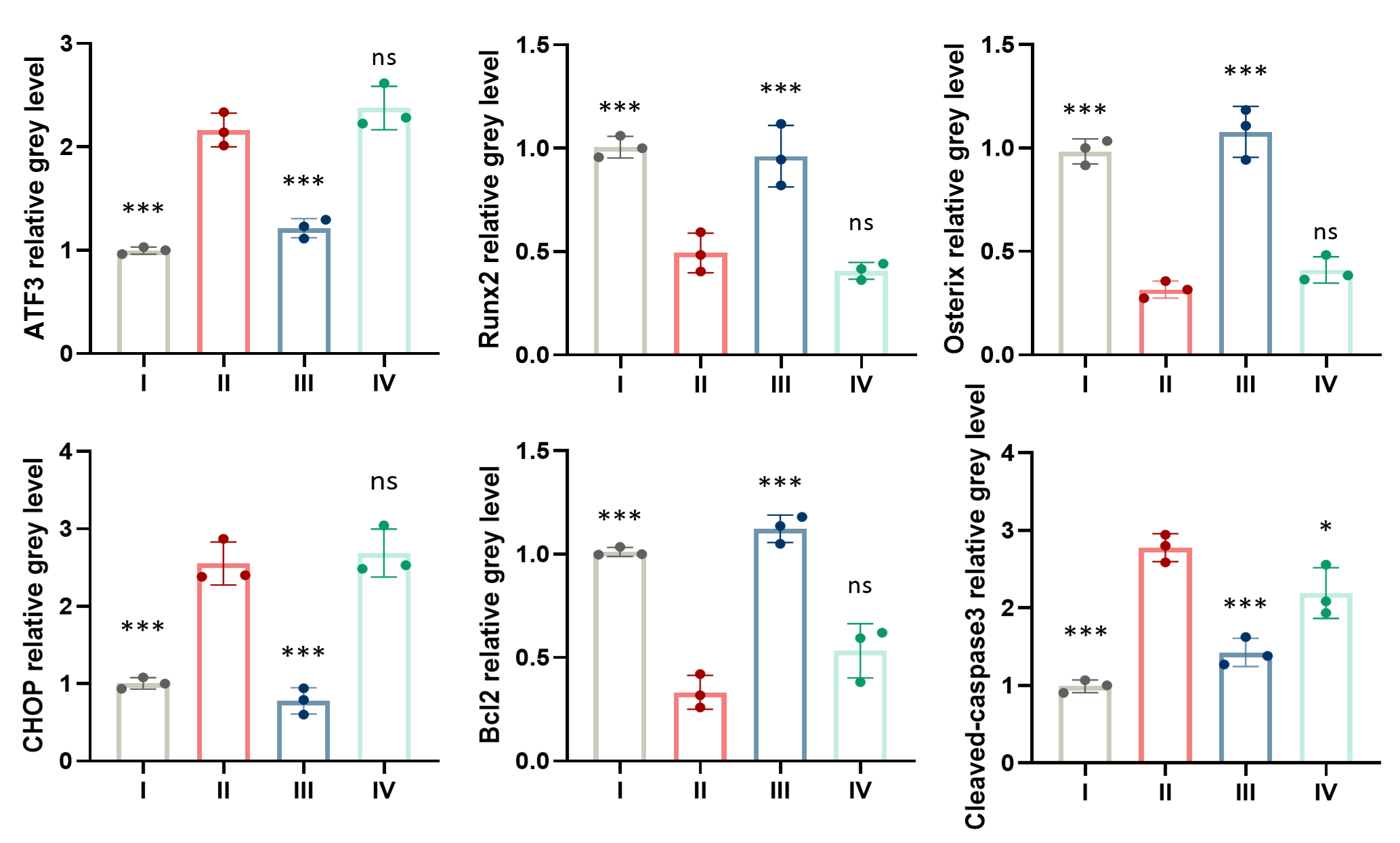


**Figure S15.** Semiquantitative analysis of the ATF3, CHOP, Runx2, Osterix, Bcl2 and cleaved-caspase3 protein levels. (I: LV-NC + Osteogenic induction, II: LV-NC + Osteogenic induction + MP, III: LV-DRD1 + Osteogenic induction + MP, IV: LV-DRD1 + ATF3 inducer + Osteogenic induction + MP, n=3 per group, ns denotes not signiﬁcant, * denotes *p* < 0.05, and *** denotes *p* < 0.001 compared to the LV-NC + Osteogenic induction + MP group).

**
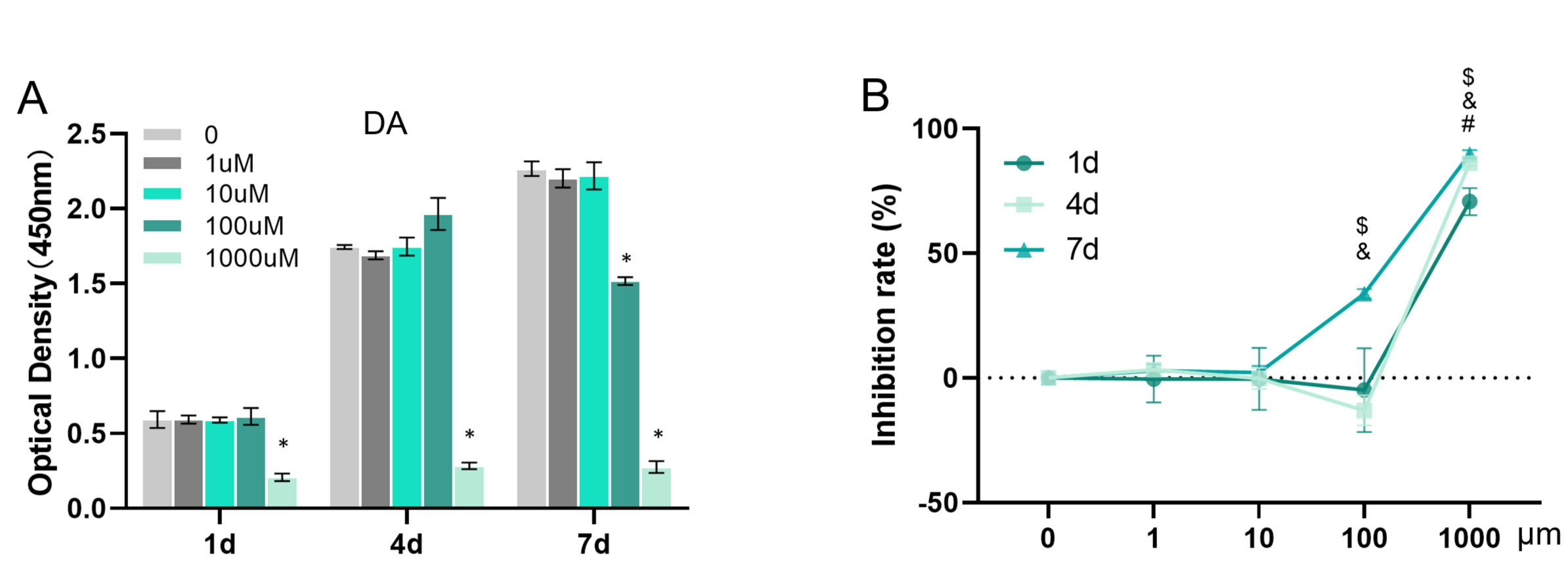
**

**Figure S16.** A) Viability of the BMSCs after incubation with DA at different concentrations (0-1000 µM) for 1, 4 and 7d (*, denotes *p* < 0.05 compared to the basal group). B) Inhibition rate of DA at different concentrations (n=5 per group, ^#^(1d), ^&^(4d), ^$^(7d) denotes *p* < 0.05 compared to the basal group).


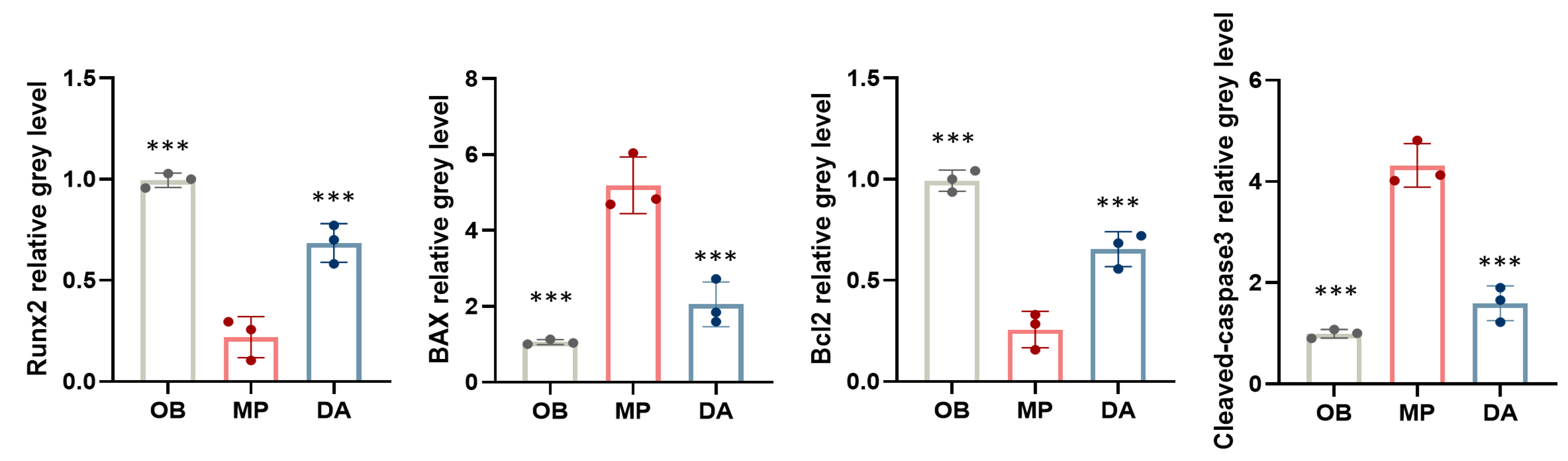


**Figure S17.** Semiquantitative analysis of the Runx2, BAX, Bcl2 and cleaved-caspase3 protein levels in osteogenesis-induced BMSCs treated with DA and then stimulated with MP (n=3 per group, *** denotes *p* < 0.001 compared to the MP group).


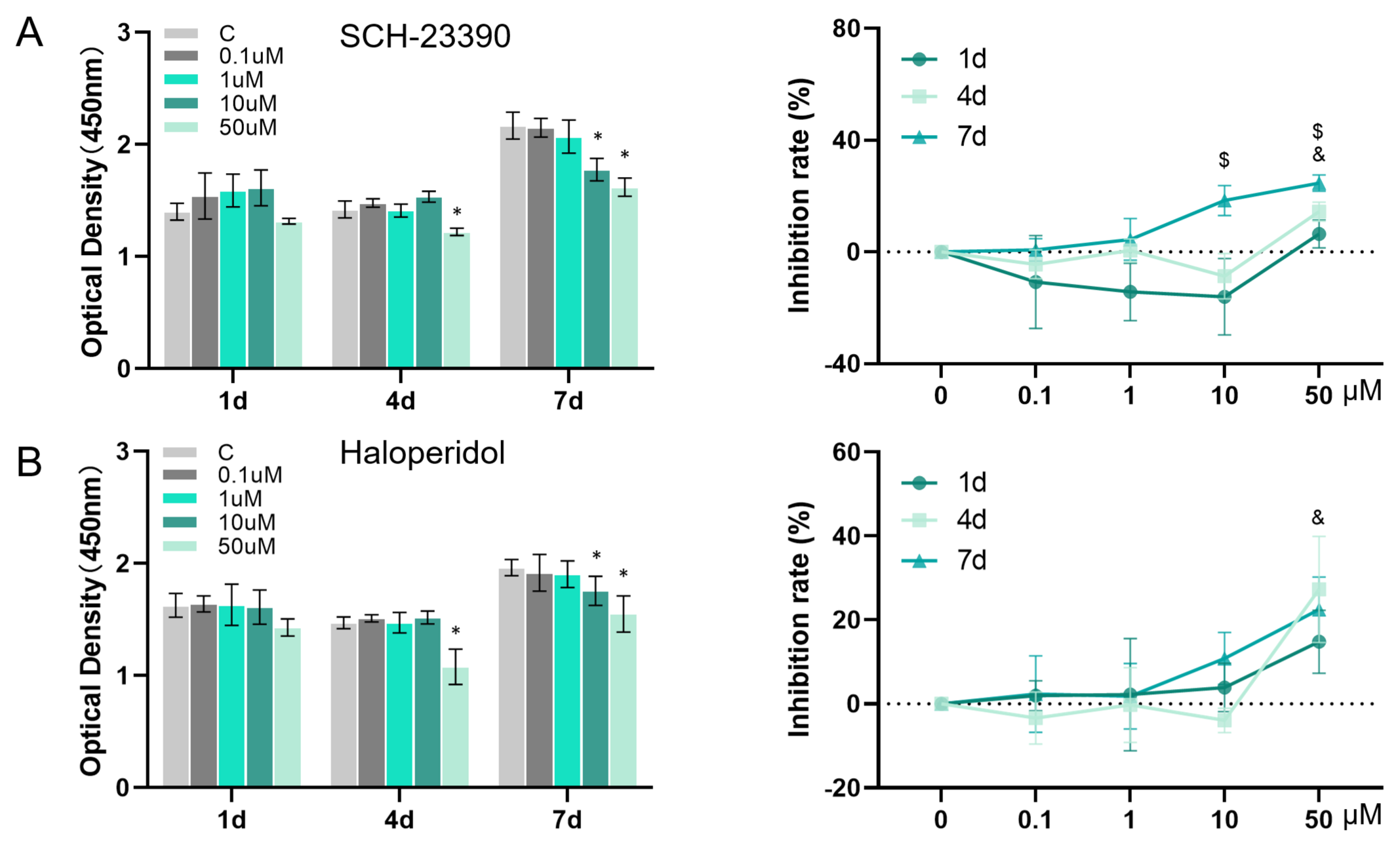


**Figure S18.** A) Viability and inhibition rate of the BMSCs after incubation with SCH-23390 at different concentrations for 1, 4 and 7d. B) Viability and inhibition rate of the BMSCs after incubation with Haloperidol at different concentrations for 1, 4 and 7d (n=5 per group, *, ^&^(4d), ^$^(7d) denotes *p* < 0.05 compared to the basal group).


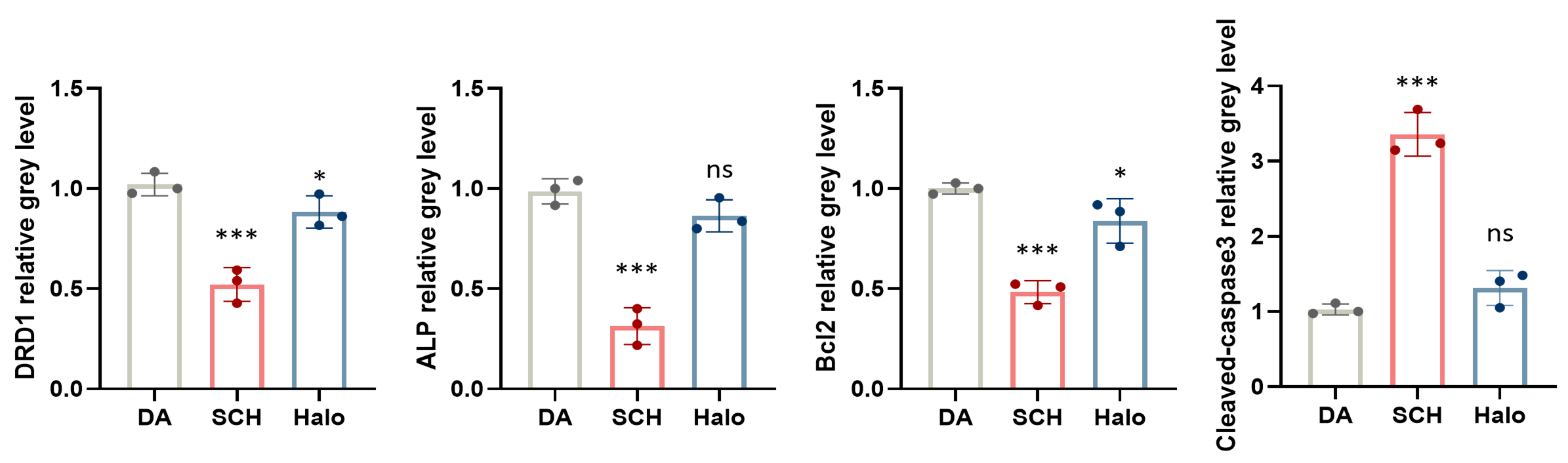


**Figure S19.** Semiquantitative analysis of the DRD1, ALP, Bcl2 and cleaved-caspase3 protein levels in in osteogenesis-induced BMSCs treated with SCH-23390 (1 μM), Haloperidol (1 μM), DA and then stimulated with MP (n=3 per group, ns denotes not signiﬁcant, * denotes *p* < 0.05, and *** denotes *p* < 0.001 compared to the DA group).

**
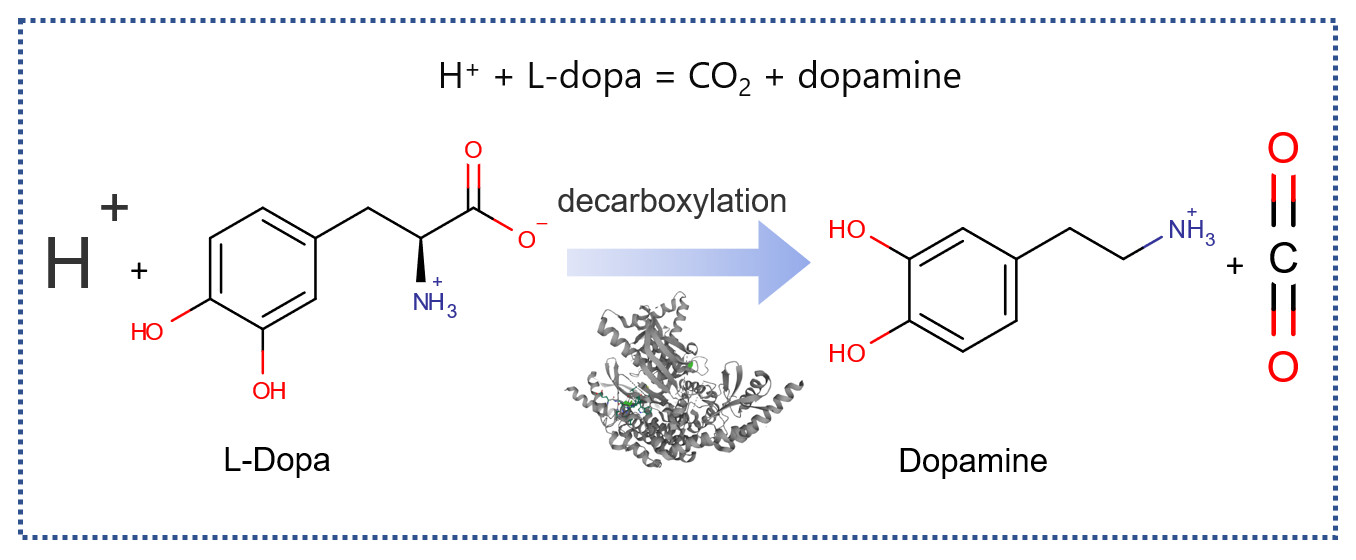
**

**Figure S20.** Chemical reaction formula of levodopa decarboxylation to dopamine by aromatic-L-aminoacid decarboxylase.

| **Table S2. Demographic data** | | | | |
| --- | --- | --- | --- | --- |
| Variables | Madopar group (n=30) | non-Madopar group (n=30) | Statistics | *p* value |
| Sex (male/female) | 7/23 | 5/25 | χ^2^=0.417 | 0.519 |
| Age (yrs) | 54.87±3.08 | 55.43±3.54 | t=-0.661 | 0.511 |
| BMI (kg/m^2^) | 26.46±4.04 | 25.42±3.87 | t=1.021 | 0.311 |
| Ficat stage (I/II/III/IV) | 8/17/5/0 | 6/18/6/0 | Z=-0.602 | 0.547 |
| Follow-up (yrs) | 5.13±2.10 | 4.83±2.09 | t=0.554 | 0.582 |

| **Table S3. Changes of Ficat stage between groups** | | | | | | | |
| --- | --- | --- | --- | --- | --- | --- | --- |
| Variables | Madopar group (n=30) | | |  | non-Madopar group (n=30) | | |
|  | pre-therapy | last follow-up | *p* value |  | pre-therapy | last follow-up | *p* value |
| Ficat stage I | 8 | 6 | 0.052 |  | 6 | 3 | <0.001 |
| Ficat stage II | 17 | 11 |  |  | 18 | 7 |  |
| Ficat stage III | 5 | 9 |  |  | 6 | 14 |  |
| Ficat stage IV | 0 | 4 |  |  | 0 | 6 |  |


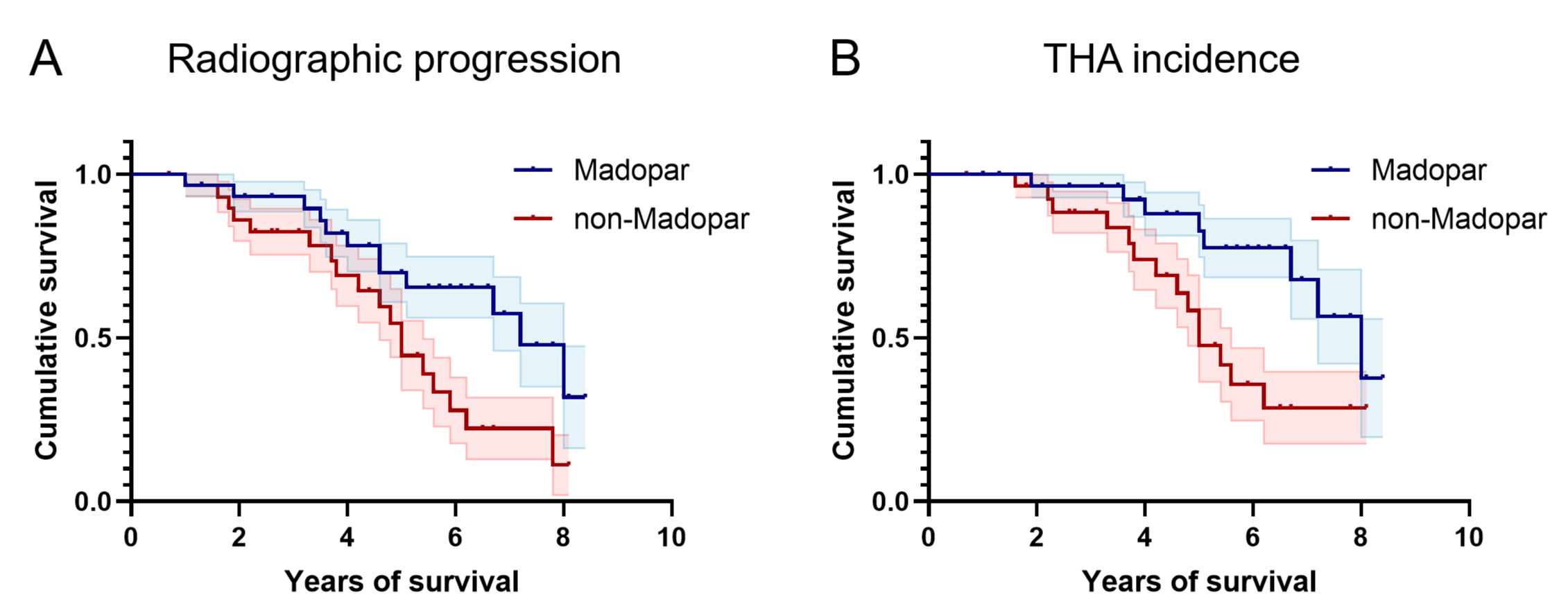


**Figure S21**. A) Kaplan–Meier survival rate according to the radiographic progression reaches Ficat stage III/IV. B) Kaplan–Meier survival rate according to the THA incidence.

| **Table S4. Changes of modified Merle d’ Aubigne score between groups** | | | | |
| --- | --- | --- | --- | --- |
| Variables | Madopar group (n=30) | non-Madopar group (n=30) | Statistics | *p* value |
| Pre-therapy | 13.50±2.00 | 14.13±2.40 | t=-1.156 | 0.252 |
| Last follow-up |  |  |  |  |
| Ficat stage I | 17.00±0.63 | 15.00±2.00 | t=2.366 | 0.050 |
| Ficat stage II | 15.91±0.70 | 13.86±1.77 | t=3.482 | 0.003 |
| Ficat stage III | 14.33±1.00 | 13.07±1.69 | t=2.252 | 0.035 |
| Ficat stage IV | 9.00±0.82 | 8.67±1.21 | t=0.478 | 0.645 |
| Total | 14.73±2.60 | 12.57±2.59 | t=3.231 | 0.002 |

**Table S5. Primer sequences used in real-time PCR**

| Gene | Forward (5’-3’) | Reverse (5’-3’) |
| --- | --- | --- |
| DRD1 | ATGGCTCCTAACACTTCTACCA | GGGTATTCCCTAAGAGAGTGGAC |
| DRD2 | ACCTGTCCTGGTACGATGATG | GCATGGCATAGTAGTTGTAGTGG |
| Runx2 | CTTCGTCAGCGTCCTATCAGTTCC | TCCATCAGCGTCAACACCATCATTC |
| Osterix | TGAGCTGGAACGTCACGTGC | AAGAGGAGGCCAGCCAGACA |
| ALP | CAGCGGGTAGGAAGCAGTTTC | CCCTGCACCTCATCCCTGA |
| OCN | GAGGCTCTGAGAAGCATAAA | AGGGCAATAAGGTAGTGAA |
| BAX | ATCTGGTTCTGCAAGCGTTTA | CCTGCTCCGAATTTGGTGAAA |
| Bcl2 | AGACGCAGCTACTGCTGTTG | CGGATCGTTTCCACCGAGAC |
| Caspase 3 | TGGTGATGAAGGGGTCATTTATG | TTCGGCTTTCCAGTCAGACTC |
| GAPDH | GCAAGTTCAACGGCACAG | CGCCAGTAGACTCCACGAC |

**Abbreviations:** DRD1: dopamine D1 receptors; DRD2: dopamine D2 receptors; Runx2: runt-related transcription factor 2; ALP: alkaline phosphatase; OCN: osteocalcin; BAX: BCL-2-associated X protein; Bcl2: B-cell lymphoma 2; Caspase 3: cysteinyl aspartate specific proteinase 3; GAPDH: glyceraldehyde 3-phosphate dehydrogenase.
